# Anti-tubulin drugs conjugated to anti-ErbB antibodies selectively radiosensitize

**DOI:** 10.1038/ncomms13019

**Published:** 2016-10-04

**Authors:** Stephen R. Adams, Howard C. Yang, Elamprakash N. Savariar, Joe Aguilera, Jessica L. Crisp, Karra A. Jones, Michael A. Whitney, Scott M. Lippman, Ezra E. W. Cohen, Roger Y. Tsien, Sunil J. Advani

**Affiliations:** 1 Department of Pharmacology, University of California San Diego, La Jolla, California 92093, USA; 2 Department of Radiation Medicine and Applied Sciences, University of California San Diego, La Jolla, California 92093, USA; 3 Department of Pathology, University of California San Diego, La Jolla, California 92093, USA; 4 Department of Medicine, University of California San Diego, La Jolla, California 92093, USA; 5 UC San Diego, Moores Cancer Center, La Jolla, California 92093, USA; 6 Department of Chemistry and Biochemistry and Howard Hughes Medical Institute, University of California San Diego, La Jolla, California 92093, USA

## Abstract

Tumour resistance to radiotherapy remains a barrier to improving cancer patient outcomes. To overcome radioresistance, certain drugs have been found to sensitize cells to ionizing radiation (IR). In theory, more potent radiosensitizing drugs should increase tumour kill and improve patient outcomes. In practice, clinical utility of potent radiosensitizing drugs is curtailed by off-target side effects. Here we report potent anti-tubulin drugs conjugated to anti-ErbB antibodies selectively radiosensitize to tumours based on surface receptor expression. While two classes of potent anti-tubulins, auristatins and maytansinoids, indiscriminately radiosensitize tumour cells, conjugating these potent anti-tubulins to anti-ErbB antibodies restrict their radiosensitizing capacity. Of translational significance, we report that a clinically used maytansinoid ADC, ado-trastuzumab emtansine (T-DM1), with IR prolongs tumour control in target expressing HER2+ tumours but not target negative tumours. In contrast to ErbB signal inhibition, our findings establish an alternative therapeutic paradigm for ErbB-based radiosensitization using antibodies to restrict radiosensitizer delivery.

## Abstract (web summary)

Drugs that sensitize tumour cells to ionizing radiation are prized because they can overcome resistance to radiotherapy. Here, the authors show that anti-tubulin drugs conjugated to cetuximab or trastuzumab can radiosensitize EGFR- or HER2-expressing tumors by increasing DNA damage and cell death due to ionizing radiation.

## Graphical abstract

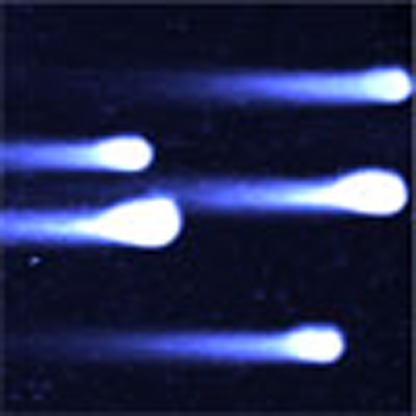

Concurrent chemotherapy and radiotherapy form the basis of curative organ sparing therapy for patients with locally advanced cancers^1^,^2^,^3^. Chemotherapy not only has intrinsic anti-tumour activity but can sometimes sensitize tumours to radiation kill. The 1990s saw multiple randomized trials unequivocally demonstrate combining cytotoxic chemotherapy (that is, cisplatin, 5-fluorouracil and taxanes) with radiotherapy to improve tumour control and patient survival^4^,^5^,^6^,^7^,^8^,^9^. However, the morbidity of such intensive regimens precludes development of more potent radiosensitizing chemotherapies^10^. Shockingly two decades later, non-targeted cytotoxic chemotherapies continue to remain the most effective approach for patients treated with concurrent chemo-radiotherapy.

To be clinically useful, radiosensitizing chemotherapies must improve the therapeutic index, that is, the level of tumour cell sensitization must be greater than surrounding normal tissue^10^,^11^,^12^. In theory, molecularly targeted radiosensitizers blocking tumour-specific pathways should increase the therapeutic index of IR by improving tumour control and decreasing side effects. Identification of ErbB (EGFR, HER2) playing a role in tumour radioresistance has led to attempts to sensitize tumours by inhibiting receptor signalling^13^,^14^,^15^,^16^,^17^,^18^,^19^,^20^. However, the efficacy of ErbB signal inhibition is limited because tumours have parallel signalling pathways circumventing the blockade^21^,^22^,^23^,^24^,^25^. Antibody drug conjugates (ADC) are emerging as a tumour targeted delivery strategy to restrict localization of drugs to tumours while sparing normal tissue^26^,^27^,^28^. ADC consist of a drug (warhead) covalently attached to an antibody recognizing a specific cell surface receptor. ADC binds to cells expressing the receptor, is then internalized by receptor-mediated endocytosis, and finally the drug is released from the antibody by the action of endolysosomal proteases. Maytansinoids and auristatins are potent anti-tubulin drugs that have been conjugated to antibodies with demonstrated clinical efficacy^29^,^30^,^31^,^32^. Importantly, we have recently discovered that monomethyl auristatin E (MMAE) is a radiosensitizer, effective at the single nM level^33^.

We hypothesized that therapeutic antibodies to ErbB receptors could direct delivery of highly potent anti-tubulin drugs in a receptor-restricted manner to selectively radiosensitize tumours. To test this hypothesis in tumour model systems, we initially synthesized two ADC in which the anti-tubulin drug monomethyl auristatin E was conjugated to cetuximab or trastuzumab (C-MMAE and T-MMAE, respectively). C-MMAE and T-MMAE bound and restricted MMAE activity and toxicity to EGFR and HER2 expressing tumour cells, respectively. Importantly while free MMAE radiosensitized indiscriminately, antibody conjugation resulted in targeted MMAE radiosensitization to EGFR or HER2 expressing tumours. To delineate the translational potential of these findings, we extended our studies to the clinically approved anti-tubulin ADC, ado-trastuzumab emtansine (T-DM1). We found that T-DM1 radiosensitized HER2 expressing tumours specifically resulting in significantly increased tumour xenograft control. On the basis of these findings, we propose antibody drug conjugate based chemo-radiotherapy paradigms designed to focus on antibody directed delivery of highly potent radiosensitizing chemotherapies as an alternative to receptor signal inhibition.

## Results

### Efficacy of anti-ErbB antibodies conjugated to Cy5 and MMAE

**Figure 1:**
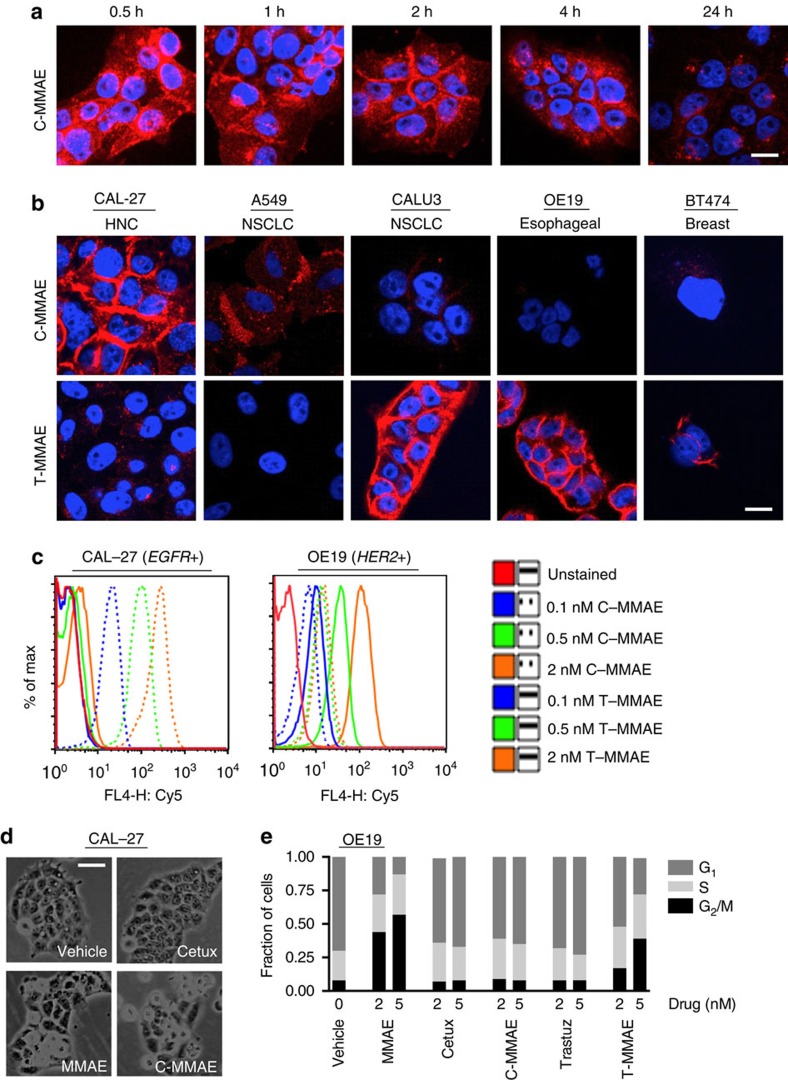
Anti-ErbB antibody MMAE conjugates bind in a receptor-dependent manner. (**a**) CAL-27 (EGFR+) cells exposed to 2 nM of Cy5-labelled C-MMAE for 30 min then incubated in drug-free media. Cells were fixed at indicated times and imaged for Cy5 fluorescence (red). Nuclei stained with DAPI (blue). Scale bar, 10 μm. Representative images of three independent experiments. (**b**) A panel of EGFR (CAL27, A549, CALU3) and HER2 (CALU3, OE19, BT474) expressing cells from diverse tumour histologies were exposed to 2 nM Cy5-labelled C-MMAE or T-MMAE for 2 h and Cy5 fluorescence (red) imaged. Nuclei stained with DAPI (blue). Scale bar, 10 μm. Representative images of three independent experiments. (**c**) Cell surface binding of Cy5-labelled C-MMAE or T-MMAE. CAL-27 and OE19 cells incubated on ice with increasing concentrations of C-MMAE or T-MMAE. of Flow cytometry assessment of Cy5 signal. Representative data of two independent experiments. (**d**) Phase contrast microscopy of CAL-27 cells treated with 2 nM MMAE, cetuximab, or C-MMAE overnight. Representative images of three independent experiments. Scale bar, 50 μm. (**e**) Cell cycle profile of OE19 cells treated with MMAE, ErbB antibodies (cetuximab or trastuzumab) or ADC (C-MMAE or T-MMAE) overnight, stained with propidium iodide and analysed by flow cytometry. Data representative of two independent experiments.

To test if ADC can restrict MMAE radiosensitization to tumours, we conjugated MMAE to cetuximab (C-MMAE) and trastuzumab (T-MMAE) and labelled them with Cy5 for tracking (Supplementary Figs 1a and 2). Cetuximab and trastuzumab were labelled at endogenous cysteines by selective reduction of the four disulfides in the hinge region and conjugation confirmed by ES-HPLC, with drug loading measured as ∼3.7 and ∼3.2 MMAE per molecule of cetuximab and trastuzumab, respectively and with ∼1 Cy5 (refs 34, 35). We used thiol-reactive maleimide derivatives of MMAE containing cathepsin-B cleavable valine–citrulline linkers that are present in the clinically approved ADC, brentuximab vedotin. We first evaluated the functionality of C-MMAE and T-MMAE. EGFR expressing CAL-27 head and neck cancer (HNC) cells were treated with C-MMAE and imaged by direct fluorescence (Fig. 1a, Supplementary Fig. 3a). By 30 min, Cy5 fluorescence localized to the cell surface and also was internalized. We then tested the specificity of C-MMAE and T-MMAE in a panel of cancer cell lines from different histologies treated with chemo-radiotherapy, HNC, non-small cell lung cancer (NSCLC) and esophageal (Fig. 1b, Supplementary Fig. 3a, Supplementary Table 1). C-MMAE bound to EGFR expressing CAL-27, A549 and CALU3 cells with decreasing affinity. T-MMAE demonstrated high affinity to the HER2 expressing cell lines CALU3, OE19 and BT474. Confocal microscopy results were validated by measuring cell surface binding of C-MMAE and T-MMAE (Fig. 1c, Supplementary Fig. 3b). CAL-27 cells bound to C-MMAE in a dose-dependent manner but not T-MMAE. In contrast, HER2 expressing cell lines, OE19, CALU3, and BT474 demonstrated dose-dependent binding of T-MMAE. We next assessed if C-MMAE and T-MMAE retained MMAE functional activity of arresting cells in G_2_/M. Morphologically, CAL-27 cells treated with either free MMAE or C-MMAE appeared identical with cells rounding up, indicative of G_2_/M (Fig. 1d). OE19 and CAL-27 cells treated with free MMAE resulted in cells accumulating in G_2_/M, the most radiosensitive phase of the cell cycle (Fig. 1e, Supplementary Fig. 4). In OE19 cells, T-MMAE blocked cells in G_2_/M in contrast to C-MMAE or antibodies alone which did not influence the cell cycle profile. In CAL-27 cells, C-MMAE resulted in G_2_/M arrest. Interestingly, our Cy5-labelled C-MMAE allowed discrimination of EGFR cell surface receptor availability that directly correlated with the potency of C-MMAE to free MMAE in EGFR expressing cell lines (Supplementary Fig. 5).

### Antibody conjugation restricts MMAE radiosensitization

**Figure 2:**
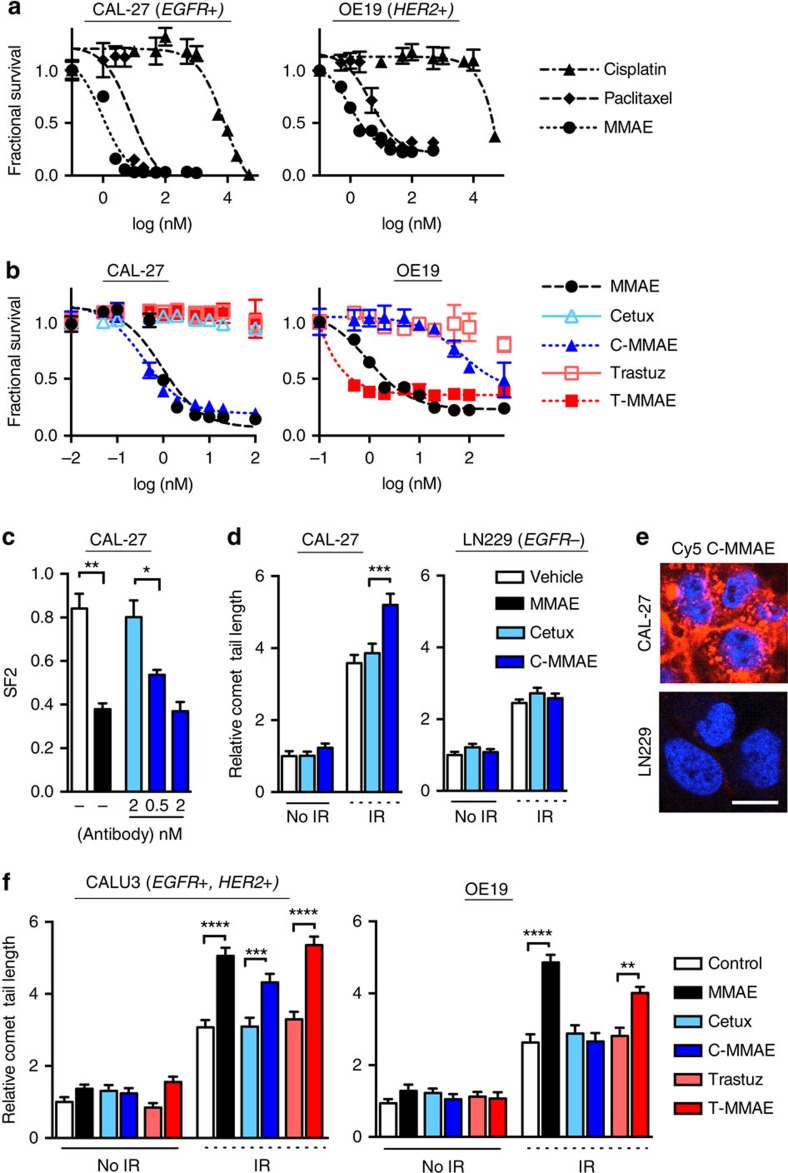
Antibody conjugated MMAE selectively radiosensitizes tumour cells. (**a**) CAL-27 (EGRF+) and OE19 (HER2+) cells were exposed to a dose range of cisplatin, paclitaxel or MMAE for 72–96 h. Cell viability normalized to vehicle treated cells and plotted as mean fractional survival±s.d. of three replicates. Data representative of three independent experiments. (**b**) CAL-27 and OE19 tumour cells were exposed to dose range of MMAE, ErbB antibody (cetuximab or trastuzumab) or ADC (C-MMAE or T-MMAE) for 72 h. Cell viability plotted as mean fractional survival±s.d. of three replicates. Data representative of three independent experiments. (**c**) Clonogenic cell survival (SF2) of CAL-27 cells treated with MMAE, cetuximab or C-MMAE overnight followed by 0 or 2 Gy. Cell viability normalized to non-irradiated cells for each drug condition and plotted as mean fractional survival±s.d. of six replicates. Data representative of two independent experiments. (**d**) CAL-27 and LN229 cells treated with cetuximab or C-MMAE overnight, irradiated with 6 Gy, and comet tail length measured by neutral comet assay. Data normalized to vehicle treated, non-irradiated cells and plotted as mean relative comet tail length±s.e.m. of >50 cells per group. Data representative of two independent experiments. (**e**) CAL-27 and LN229 cells exposed to 2 nM Cy5-labelled C-MMAE for 2 h and Cy5 fluorescence (red) imaged. Nuclei stained with DAPI (blue). Scale bar, 10 μm. (**f**) CALU3 (EGFR+, HER2+) and OE19 cells treated with free MMAE, ErbB antibodies (cetuximab or trastuzumab) or ADC (C-MMAE or T-MMAE) overnight, irradiated with 6 Gy, and comet tail length measured using neutral comet assay. Comet tail length normalized to vehicle treated, non-irradiated cells and plotted as mean relative comet tail length±s.e.m. of >50 cells per group. Data representative of two independent experiments. All statistical significances were calculated using one-way ANOVA with Tukey's multiple comparisons test. *P<0.05, **P<0.01, ***P<0.001. ****P<0.0001.

Key advantages driving the clinical development of MMAE are its potency and its ability to be tumour targeted through antibody conjugation^27^,^28^. In cell lines we tested, MMAE was more potent compared with standard cytotoxic chemotherapies that are used concurrently with radiotherapy in patients, that is, cisplatin and paclitaxel (Fig. 2a)^33^. Importantly, C-MMAE or T-MMAE restricted MMAE cytotoxicity to EGFR or HER2 expressing cells, respectively, and were much more potent than antibodies alone (Fig. 2b). Since concurrent chemo-radiotherapy is standard of care in HNC, NSCLC and esophageal cancers, we tested whether ADC restricted MMAE radiosensitization in cell lines of these histologies^1^,^2^,^3^. Following IR, cells die predominantly as a result of mitotic catastrophe that can be measured by clonogenic survival^11^. CAL-27 cells were treated overnight with MMAE, cetuximab or C-MMAE and then irradiated. The surviving fraction following 2 Gy decreased in cells treated with free MMAE or C-MMAE compared with vehicle or cetuximab-treated cells (Fig. 2c). At these lower drug concentrations, cetuximab had no radiosensitizing effect. Mechanistically, IR induces cell death by causing DNA double strand breaks, which can be measured by neutral comet assay^11^. Irradiation of vehicle-treated CAL-27 cells resulted in a 3.6-fold increase in comet tail length that was significantly increased by 0.5 nM C-MMAE but not by 5 nM cetuximab (Fig. 2d). Importantly, C-MMAE restricted MMAE radiosensitization. In low EGFR binding LN229 cells (Fig. 2e, Supplementary Fig. 6), irradiation resulted in a 2.5-fold increase in comet tail length that was not appreciably increased by 20 nM cetuximab or C-MMAE (Fig. 2d). Interestingly in irradiated CALU3 cells, 2 nM of free MMAE, C-MMAE or T-MMAE all resulted in increased comet tail length to a similar degree when compared with vehicle or 20 nM antibody-treated cells (Fig. 2f). This result is concordant with cytotoxicity data, which demonstrated C-MMAE and T-MMAE were equally cytotoxic in CALU3 cells (Supplementary Fig. 7). In irradiated HER2+ OE19 cells, 2 nM free MMAE or T-MMAE further increased comet tail length compared vehicle, 20 nM antibody or 2 nM C-MMAE treated cells (Fig. 2f).

### Maytansinoids radiosensitize and can be HER2 targeted

**Figure 3:**
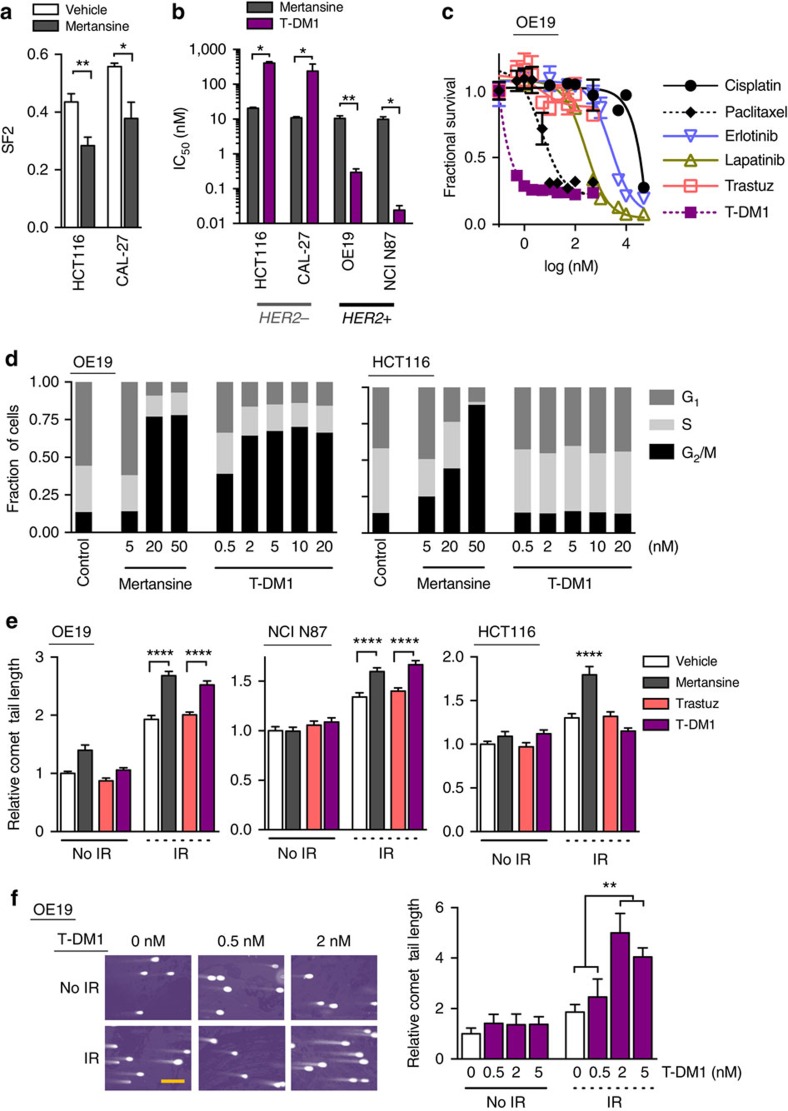
Clinical grade anti-tubulin ADC T-DM1 radiosensitizes HER2+ tumours. (**a**) Clonogenic cell survival (SF2) of cells treated with 5 nM mertansine overnight followed by 2 Gy. Cell viability normalized to non-irradiated cells for each drug condition and plotted as mean fractional survival±s.d. of five replicates. Data representative of two independent experiments. (**b**) IC_50_ of mertansine and T-DM1 in cells based on HER2 expression. Cells treated with a dose range of drug for 72–96 h and viability measured, IC_50_ calculated and plotted as mean±s.d. of three independent experiments. (**c**) Cytotoxicity of clinically used radiosensitizers. OE19 cells were exposed to a dose range of drugs, viability normalized to vehicle treated cells and plotted as mean fractional survival±s.d. of three replicates. Data representative of three independent experiments (**d**) Cell cycle profile of OE19 and HCT116 cells treated with T-DM1 overnight, stained with propidium iodide and analysed by flow cytometry. Data representative of two independent experiments. (**e**) Neutral comet assay of HER2+ and HER2− cells treated with 20 nM mertansine, trastuzumab or T-DM1 overnight and irradiated with 2 Gy. Comet tail length was normalized to vehicle treated, non-irradiated cells and plotted as relative comet tail length±s.e.m. of >50 cells per group. Data representative of two independent experiments. (**f**) Representative images of comet tails and quantification of relative comet tail length of OE19 cells treated with 0–5 nM of T-DM1 and irradiated. Comet tail length was normalized to vehicle treated, non-irradiated cells and plotted as relative comet tail length±s.e.m. of >50 cells per group. Scale bar, 50 μm. All statistical significances were calculated using one-way ANOVA with Tukey's multiple comparisons test. *P<0.05, **P<0.01, ****P<0.0001.

Similar to the auristatins (for example, MMAE), maytansinoids (for example, mertansine) are another potent class of anti-tubulins^29^. Importantly, mertansine forms the warhead of the trastuzumab ADC T-DM1, and is attached to lysine residues of trastuzumab through a thiol ether linker (Supplementary Fig. 1b). T-DM1 (Kadcyla) has shown efficacy in HER2 expressing metastatic breast cancer patients^29^,^30^. Given our findings that T-MMAE selectively radiosensitized HER2 expressing cancer cells, we decided to evaluate the more immediate clinical potential of our strategy by testing if T-DM1 radiosensitized tumours in a HER2+ selective manner. First, we tested if the maytansinoid warhead of T-DM1 was a bona fide radiosensitizer. HCT116 or CAL-27 cells were treated overnight with mertansine and then irradiated with 2 Gy. In both cell lines, the surviving fraction following 2 Gy decreased further in cells treated with free mertansine compared with vehicle (Fig. 3a). Irrespective of HER2 expression, the IC_50_ of mertansine was fairly consistent across cell lines ∼10 nM (Fig. 3b). In contrast, T-DM1 potency directly correlated with HER2 expression. In HER2+ cell lines (OE19 and NCI N87 (gastric cancer)) the IC_50_ of T-DM1 was <1 nM but was >100 nM in HER2- cells(HCT116 and CAL-27). From a translation point of view, T-DM1 was a more potent cytotoxic drug in HER2+ OE19 cells compared with clinically used radiosensitizers that are either cytotoxic (paclitaxel and cisplatin) or targeted to HER2 (trastuzumab and lapatinib) or EGFR (erlotinib) (Fig. 3c). However in HER2- CAL-27 cells, paclitaxel was more potent than T-DM1 (Supplementary Fig. 8). Functionally, mertansine increased the accumulation of both OE19 and HCT116 cells in the radiosensitive G_2_/M phase of the cell cycle (Fig. 3d). While T-DM1 also blocked OE19 cells in G_2_/M, the cell cycle profile of HCT16 cells treated with T-DM1 resembled vehicle-treated cells. Next, we tested if T-DM1 would restrict mertansine mediated radiosensitization in a HER2-dependent manner. Unconjugated mertansine (20 nM) radiosensitized by increasing IR induced DNA double-strand breaks in both HER2+ and HER2− cell lines (Fig. 3e). Importantly, 20 nM T-DM1 resulted in restricted radiosensitization of HER2+ cells. As with T-DM1's increased potency and ability to arrest cells in G_2_/M confined to HER2+ cells, trastuzumab conjugation reversed mertansine's ability to increase IR induced DNA double-strand breaks, making it appear inert to irradiated non-HER2 expressing cells. At these dose levels, trastuzumab did not result in any significant radiosensitization. Moreover, doses as low as 2 nM T-DM1 radiosensitized OE19 cells (HER2+) as they accumulated in the G_2_/M phase of the cell cycle (Fig. 3d,f).

### ADC more effectively radiosensitize tumours than free drugs

**Figure 4:**
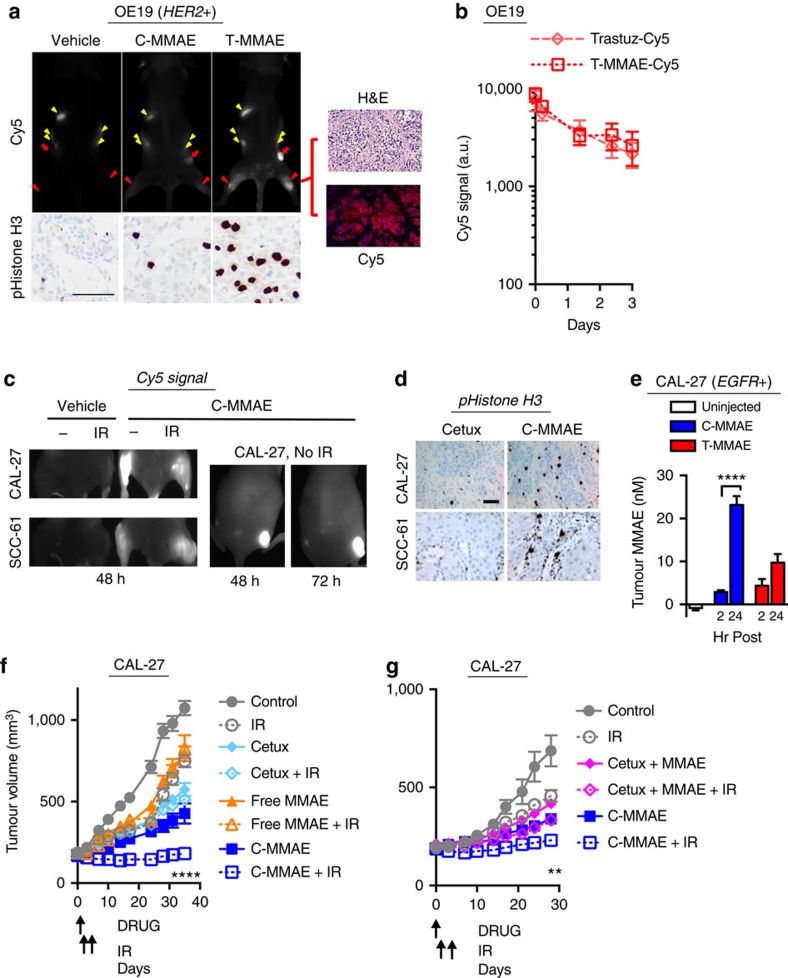
Antibody conjugated MMAE selectively targets tumours and increases efficiency of tumour regression in combination with IR. (**a**) OE19 tumour xenografts grown in the flank and bilateral thighs (location indicated by three red arrows) of mice and 0.5 nmol of Cy5-labelled C-MMAE or T-MMAE (∼4 and ∼3.9 mg kg^−1^, respectively) intravenously injected. Tumours imaged 48 h later for Cy5 fluorescence (upper left). Yellow arrowheads indicate gut auto-fluorescence. Tumour xenografts were harvested and stained for pHistone H3 (bottom left). From T-MMAE injected mice, tumours were H&E stained and imaged for Cy5 fluorescence (right). Scale bar, 50 μm. Representative imaging of two mice per group. (**b**) Mice were injected with Cy5-labelled T-MMAE, blood collected, Cy5 fluorescence measured and plotted as mean±s.d. of four mice. (**c**) EGFR+ tumour xenografts grown in the bilateral thighs of athymic nude mice (left), 0.5 nmoles of Cy5-labelled C-MMAE (∼4 mg kg^−1^) injected, 24 h later right tumour bearing thigh irradiated with 3 Gy and mice imaged 24 h post IR for Cy5 fluorescence. Non-irradiated, mice bearing unilateral CAL-27 tumour xenografts (right), 0.5 nmol of Cy5-labelled C-MMAE (∼4 mg kg^−1^) injected and mice imaged at 48 and 72 h for Cy5 fluorescence. Representative imaging of three mice per group. (**d**) EGFR+ tumour-bearing mice were injected with cetuximab or C-MMAE, harvested 24 h later and stained for pS10 histone H3. Scale bar, 50 μm. Representative imaging of three mice per group. (**e**) CAL-27 tumour-bearing mice were injected with 2 nmol of C-MMAE or T-MMAE (∼15.9 and ∼15.4 mg kg^−1^, respectively) and harvested. Tumour drug concentrations quantitated LC-MS/MS and plotted as mean±s.e.m. of four tumours. Statistical significance was calculated using one-way ANOVA with Tukey's multiple comparisons test. (**f**,**g**) Mice bearing CAL-27 tumours injected on day 0 with indicated drugs on day 0 and 3 Gy given on days 1, 2. Tumours were measured and plotted as mean tumour volume±s.e.m. of ≥8 tumours per group. Statistical significances were calculated using two-way ANOVA with Tukey's multiple comparisons test. **P<0.01, ****P<0.0001.

We then tested the efficacy of combining ErbB targeted delivery of anti-tubulins with IR in tumour xenografts. We first focused on our MMAE synthesized cetuximab and trastuzumab ADC since Cy5 labelling allows for *in vivo* ADC visualization, tracking and serves as a surrogate for tumour drug delivery following intravenous injection in mice. Mice bearing OE19 tumours were intravenously injected with Cy5-labelled C-MMAE or T-MMAE (Fig. 4a). In these HER2+ tumours, T-MMAE demonstrated greater Cy5 signal accumulation within tumours compared with C-MMAE. MMAE target ‘hit' was validated by staining tumours for the G_2_/M marker, pS10 of Histone H3. T-MMAE resulted in increased pS10 of Histone H3 in tumours while cetuximab conjugation blocked MMAE anti-tubulin activity. Sectioned tumour xenografts demonstrated T-MMAE Cy5 fluorescence localized to areas of tumour cells and not stroma, an important pre-requisite for radiosensitizers to improve the therapeutic ratio. T-MMAE pharmacokinetics, measured by loss of Cy5 fluorescence in the blood was comparable to that of similar Cy5-labelled antibody alone (Fig. 4b)^36^. Conversely in EGFR expressing tumours HNC (CAL-27, SCC-61, SCC-35 and SQ-9G), NSCLC (A549) and colorectal (HCT-116), tumour xenografts showed Cy5 signal accumulation for up to 72 h after C-MMAE injection while tumours from low EGFR LN-229 did not (Fig. 4c, Supplementary Fig. 9). Irradiation of tumour xenografts did not appreciably influence C-MMAE accumulation in tumours. EGFR+ tumour xenografts from mice treated with C-MMAE showed increased accumulation in G_2_/M by pS10 Histone H3 staining compared with cetuximab, verifying delivery of active MMAE to tumours (Fig. 4d). Importantly, tumour MMAE concentrations from CAL-27 xenografts injected with C-MMAE or T-MMAE confirmed restriction of released drug to EGFR+ cells by cetuximab conjugation as opposed to trastuzumab conjugation (Fig. 4e). Next, we assessed the efficacy of anti-ErbB antibody MMAE conjugates in combination with IR on tumour regression. Mice with CAL-27 tumours were treated with vehicle, free MMAE, cetuximab or C-MMAE on day 0 (Fig. 4f). Cetuximab and C-MMAE were injected at a dose of 0.5 nmol (∼3.6 and ∼4 mg kg^−1^, respectively). Since an average of four molecules of MMAE were conjugated to each antibody molecule, a dose of 2 nmol of free MMAE was injected to maintain MMAE dose equivalence to C-MMAE. Because C-MMAE persisted in murine xenografts for up to 72 h (Fig. 4c), 3 Gy was given on days 1 and 2. In non-irradiated tumours, both free MMAE and cetuximab alone delayed tumour growth, which was further slowed by C-MMAE. Importantly, delivering IR with C-MMAE significantly increased tumour growth delay, *P*<0.0001 compared with all other experimental groups by day 35 post initiation of treatment (Supplementary Table 2). To specifically ascertain the advantage of using ADC in combination with IR, we tested C-MMAE compared with co-administered free MMAE and cetuximab (Fig. 4g). Interestingly in irradiated groups, mice receiving C-MMAE had significantly smaller tumours than those receiving co-administered cetuximab and free MMAE, *P*<0.05 by day 28 (Supplementary Table 3). In both experiments, mice tolerated therapies well as measured by weekly weights (Supplementary Fig. 10). These results reveal the advantages of using antibody conjugated MMAE as a radiosensitizer as opposed to the combination of antibodies with cytotoxic drugs on tumour control and is line with recent negative results from phase III clinical trials^37^,^38^.

### T-DM1 and IR prolong HER2+ but not HER2− tumour control

**Figure 5:**
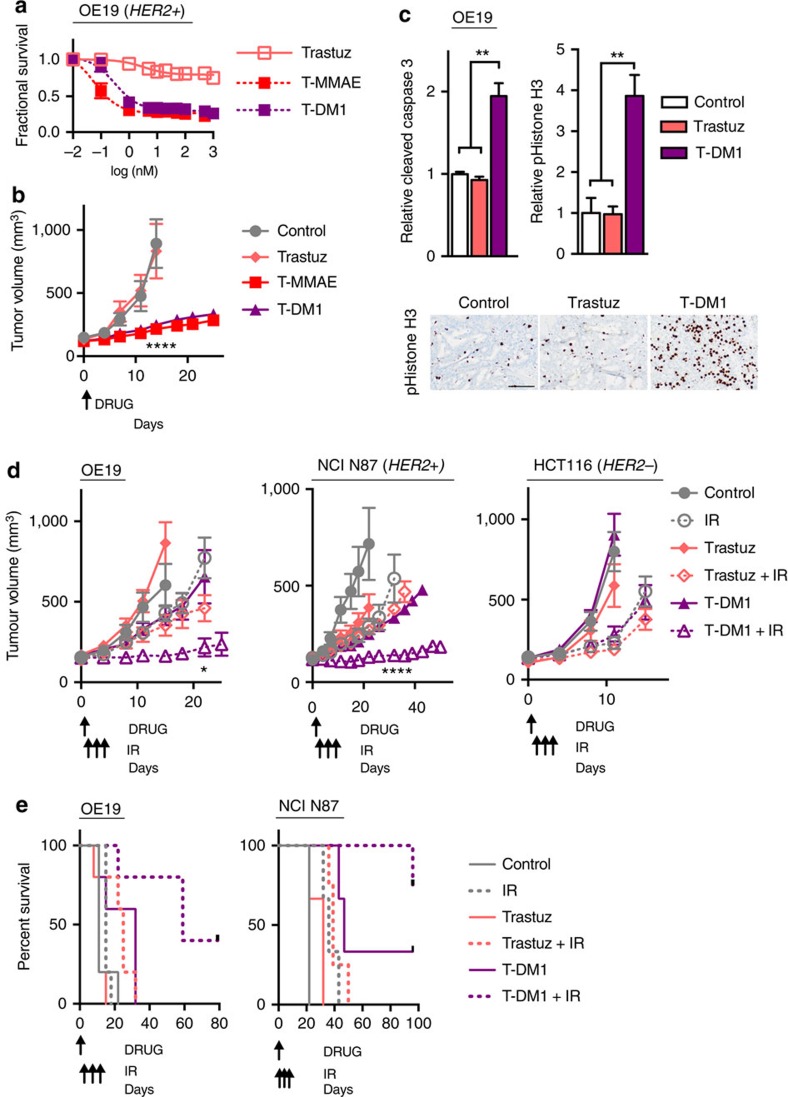
T-DM1 combined with IR results in long-term HER2+ tumour control. (**a**) OE19 cells were exposed to trastuzumab, T-MMAE or T-DM1 for 96 h. Cell viability normalized to vehicle-treated cells and plotted as mean fractional survival±s.d. of three replicates. Data representative of three independent experiments. (**b**) Mice bearing OE19 tumour xenografts were i.v. injected on day 0 with 1 nmol trastuzumab, T-DM1 or T-MMAE (∼7.3, ∼7.4 and ∼7.7 mg kg^−1^, respectively). Tumours were measured twice a week and plotted as mean tumour volume±s.e.m. of 10 tumours per group. Statistical significances were calculated using two-way ANOVA with Tukey's multiple comparisons test. (**c**) Tumour from OE19 tumours harvested 48 h after trastuzumab or T-MMAE and stained for cleaved caspase 3 or p-Histone H3 by IHC. Data were normalized to untreated, control tumours and plotted as mean±s.e.m. of ≥3 replicates. Statistical significance was calculated using one-way ANOVA with Tukey's multiple comparisons test. Representative images of pHistone H3 shown. Scale bar, 75 μm. (**d**) Mice bearing OE19 or NCI N87 (HER2+) or HCT116 (HER2−) tumour xenografts were i.v. injected on day 0 with 0.25 nmol trastuzumab or T-DM1 (∼1.8 and ∼1.9 mg kg^−1^, respectively). IR-treated mice were given 2.5 Gy on days 1, 2 and 3. Tumours were measured twice a week and plotted as mean tumour volume±s.e.m. of ≥4 tumours per group. Statistical significances were calculated using two-way ANOVA with Tukey's multiple comparisons test. (**e**) Survival curve of HER2+ treated tumour xenografts of ≥2 mice per group . Statistical significances were calculated using log-rank (Mantel–Cox) test. *P<0.05, **P<0.01, ****P<0.0001.

Given these findings with C-MMAE and T-MMAE in EGFR+ and HER2+ tumour xenografts, we next evaluated T-DM1 since it is already clinically approved and its safety established in women with metastatic breast cancer^30^. First, we compared the potency of auristatins and maytansinoids conjugated to trastuzumab. In HER2+ OE19 and CALU3 cells, T-MMAE and T-DM1 had similar potency (Fig. 5a, Supplementary Fig. 11). In OE19 HER2+ tumour xenografts, a single dose of 1 nmol of either T-MMAE or T-DM1 (∼7.7 and ∼7.4 mg kg^−1^, respectively) were equally efficacious in slowing tumour growth compared with control or trastuzumab by day 14 (Fig. 5b, Supplementary Table 4). While T-DM1 increased apoptosis, it also increased the number of cells in radiosensitive G_2_/M phase of the cell cycle, as measured by pS10 Histone H3 (Fig. 5c). Given the equivalence of T-MMAE and T-DM1 in cell culture and tumour xenografts, we then focused on optimizing dosing of T-DM1 and IR to determine if combining the two treatments would produce long term tumour control specifically in HER2+ tumour xenografts. We dose reduced T-DM1 to 0.25 nmol (∼1.9 mg kg^−1^) delivered once (day 0). On the basis of our above results with our Cy5-labelled ADC, ADC localized within tumours for up to 72 h. Therefore, we delivered 2.5 Gy of IR on days 1, 2 and 3 after T-DM1 injection. In both OE19 and NCI N87 HER2+ tumour xenografts, this regimen resulted in significantly prolonged tumour regression (Fig. 5d), increased doubling time (Table 1, Supplementary Table 5), while mice maintained their weight (Supplementary Fig. 10). By day 22 in OE19 and day 32 in NCI N87 tumour xenografts, T-DM1 combined with IR was superior to all other treatment regimens (Supplementary Tables 6 and 7). In stark contrast, HER2- HCT116 tumour xenografts showed no benefit of adding T-DM1 to IR (Fig. 5d, Table 1, Supplementary Table 8). Of potential clinical value, long term tumour control was observed when T-DM1 was combined with IR in HER2+ tumours (Fig. 5e, Supplementary Table 9).

## Discussion

Unresectable, locally advanced cancers continue to pose a therapeutic challenge^1^,^2^,^3^. The most effective chemo-radiotherapy strategies to date integrate non-targeted cytotoxic chemotherapies^3^,^4^,^5^,^6^,^7^,^8^,^9^,^39^,^40^. However, such therapeutic intensification increases normal tissue toxicities often precluding further radiotherapy or chemotherapy dose escalation^10^. To mitigate treatment related side effects and allow for more potent radiosensitizing chemotherapies, tumour targeted radiosensitization approaches are required. While targeted drug therapies have advanced for certain cancer patients with specific mutations, outside of cetuximab none have demonstrated unequivocal clinical utility with radiotherapy when compared directly with non-targeted cytotoxic chemotherapies^12^,^13^,^41^,^42^. Frustratingly, adding cetuximab to cytotoxic chemo-radiotherapy in NSCLC or HNC patients failed to improve outcomes^37^,^38^.

While inhibiting ErbB signalling is an appealing approach to radiosensitize tumours, attempted blockage of receptor tyrosine kinase signalling results in activation of bypass pathways, that is the ‘whack a mole' problem^21^,^24^,^25^. To overcome this, we propose an alternative ErbB mediated radiosensitization paradigm based on ErbB directed ADC that is more potent and potentially superior to signal inhibition. We initially evaluated the auristatin class (MMAE) of anti-tubulins as ADC-based radiosensitizers for three reasons. First, it is the ‘warhead' of brentuximab vedotin, a clinically used ADC for CD30 lymphomas that is attached through a cathepsin B sensitive and self-immolative linker. Moreover, this allowed for direct measurement of tumour drug delivery (Fig. 4)^35^. Second, we have previously demonstrated that in addition to its cytotoxic activity, MMAE is a potent radiosensitizer^33^. Finally, Cy5 labelling during ADC synthesis allowed for non-invasive imaging of ADC localization and served as a marker for antibody targeting which was validated by tumour drug delivery and activity of blocking cells in G_2_/M. Our synthesized ADC showed tumour accumulation of T-MMAE or C-MMAE in a receptor-restricted manner which resulted in tumour selective MMAE radiosensitization. Given our findings with C-MMAE and T-MMAE, we extended our studies to the clinically approved ADC, T-DM1. As with auristatins, we found that the maytansinoid class of anti-tubulins also were potent radiosensitizers as either free drug or as the trastuzumab ADC, T-DM1 (Figs 3 and 5).

Delivering ADC with IR has several advantages that can result in improved patient outcomes: (1) Combinatorial therapy attacks cancer cells by multiple mechanisms decreasing the risk of tumour resistance emerging. (2) Instead of higher individual doses of a single agent to achieve effective tumour kill, combinatorial therapy allows for dose reduction of each individual modality thereby decreasing the toxicities inherently associated with each therapy. (3) concurrent delivery of full dose chemotherapy and radiotherapy allow for attacking not only known local disease but also potential micrometastases. (4) Ideally, chemotherapies used in conjunction with radiotherapy will be highly potent alone, synergize with IR, and be spatially targeted to tumours and not normal tissue. (5) The precise timing with which IR can be delivered is valuable for defining the temporal window(s) when radiosensitization should be maximal. Auristatin and maytansinoid based ADC fulfill these criteria. Currently, there are only two indications for which ADC has shown clinical value, CD30 lymphomas and HER2 metastatic breast cancer^30^,^31^. Our studies and in particular those involving T-DM1, suggest integrating ADC in the curative setting for locally advanced solid tumours. HER2 overexpression occurs in a proportion of lung, esophageal, gastric and bladder cancers, which are treated with concurrent chemo-radiotherapy^2^,^3^,^43^,^44^. On the basis of our findings, T-DM1 provides potent and tumour selective radiosensitization that warrants speedy clinical evaluation and may help expedite the evolution of tumour radiosensitization from decades old non-targeted cytotoxins to a biomarker-driven tumour-targeted paradigm.

## Methods

### Cells and reagents

All cell lines used in these studies are summarized in Supplementary Table 1. Human HNC (CAL-27, SCC-25), NSCLC (A549, CALU3), colorectal (HCT-116), gastric (NCI N87), glioma (LN229) and breast (BT474) cancer cell lines were obtained from American Type Culture Collection. Human esophageal cancer line OE19 was obtained from Sigma-Aldrich. Human HNC SCC-35, SCC61 and SQ-9G was kindly provided from Ralph Weichselbaum, University of Chicago. CAL-27, A549 and HCT116, cells were cultured in DMEM supplemented with 10% FBS. SCC-61 was cultured in DMEM/F12 supplemented with 20% FBS and 400 ng ml^−1^ hydrocortisone. OE19 and NCI N87 cells were cultured in RPMI supplemented with 10% FBS. CALU3 cells were cultured in MEM supplemented with 10% FBS. On receipt, each cell line was expanded, cryopreserved as low passage stocks and routinely tested for mycoplasma. Cisplatin (Enzo Biosciences), paclitaxel (Sigma), MMAE (Concortis) and mertansine (Abcam) were reconstituted in DMSO. Clinical-grade erlotinib, lapatinib, cetuximab, trastuzumab, ado-trastuzumab emtansine (T-DM1) were obtained from UCSD Moores Cancer Center pharmacy.

### Synthesis of MMAE and Cy5 anti-ErbB antibodies conjugates

A solution (1 ml, 2 mg ml^−1^) of cetuximab (Erbitux, ImClone) or trastuzumab (Herceptin, Roche) was treated with sodium bicine buffer (100 μl, 1 M pH 8.3) and sodium diethylenetriaminepentaacetic acid (10 μl, 100 mM pH 7). Following reduction with four equivalents of tris(carboxyethyl)phosphine (TCEP) at 37 °C for 2 h, the solution was added to four equivalents of maleimidocaproyl-valine-citrulline-PABA-MMAE (MC-VC-MMAE)^33^. After 30 min at room temperature, Cy5-maleimide (2 equivalents) was added and after a further 30 min, gel-filtered (Sephadex G25, 0.6 g) eluting with PBS. Following centrifugal concentration (Centricon 30 kDa MWCO) to 500 μl, the concentrations of antibody and Cy5 were determined by absorbance using extinction coefficients of 210,000 M^−1^ cm^−1^ (cetuximab) or 225,000 M^−1^ cm^−1^ (trastuzumab) at 280 nm and 12,500 M^−1^ cm^−1^ and 250,000 M^−1^ cm^−1^ at 280 and 650 nm, respectively, for Cy5. Hydrophobic interaction chromatography (HIC) of a reaction sample after labelling with MMAE revealed nine peaks corresponding to antibody modified with 0–8 MMAE derivatives if up to four disulfides are reduced by TCEP per antibody^34^ (Supplementary Fig. 2a,c). Subsequent Cy5-maleimide labelling gave 650 nm absorbance to each peak apart from that labelled with eight MMAE as no cysteines are available for further conjugation (Supplementary Fig. 2b,d). Drug loading was measured by denaturing reverse-phase HPLC of the reaction mix before addition of Cy5 maleimide, following reduction of any remaining intersubunit disulfides with 50 mM DTT for 30 min (ref. 45). Peaks corresponding to light or heavy chains^46^,^47^ with 0–3 MMAE were identified by electro-spray mass spectroscopy (Supplementary Fig. 2e–h) and peak areas at 280 nm were integrated and weighted to calculate the drug loading^26^. Modified light chain (L1) and unmodified H chain (H0) were not resolved for trastuzumab so MMAE loading is an underestimate. No free MC-VC-MMAE was detected by HPLC following gel filtration. This conjugation chemistry that we utilized has subsequently been shown to undergo a slow retro-Michael reaction resulting in loss of the MMAE-linker from the antibody in the circulation and potential off-target toxicity. Modifications have been devised to decrease premature release of the warhead but have yet to be clinically approved, so we retained the established linker^48^.

### Cy5 fluorescence imaging

Cells were exposed to Cy5-labelled cetuximab-MMAE or trastuzumab-MMAE for 30 min in media with 1% serum. Cells were then washed with PBS and incubated in media with 10% serum. At indicated times, cells were fixed in 4% paraformaldehyde and then stained with DAPI. Cells were imaged using a Nikon A1R confocal microscope.

### ADC cell binding

Cells were collected and resuspended in cold PBS with 5% BSA. Cy5-labelled cetuximab-MMAE or trastuzumab-MMAE was added to the cells at indicated concentrations for 15 min on ice. Cells were washed, resuspended in PBS with 5% BSA and 0.5 μg ml^−1^ propidium iodide, and analysed by flow cytometry.

### Cell cycle

Cells were treated with MMAE, mertansine, cetuximab or cetuximab-MMAE, trastuzumab, trastuzumab-MMAE or T-DM1 for 24 h and then fixed in methanol. Cells were treated with RNAse, stained with propidium iodide (PI) and analysed by FACS using FloJo software.

### Alamar blue assay

Cells were plated in 96-well plates and exposed to a range of concentrations of MMAE, mertansine, cisplatin or paclitaxel, cetuximab, cetuximab-MMAE, trastuzumab-MAME or T-DM1 for 72 h. Alamar Blue (resazurin) was added to the cells and allowed to incubate for 2–4 h at 37 °C. Plates were analysed using a plate reader with fluorescence measured at 560 nm.

### Clonogenic assay

Cells were treated with MMAE, mertansine, cetuximab or cetuximab-MMAE for 24 h and then irradiated with 2 Gy. Following IR, cells were counted, re-plated at varying cell numbers in drug-free media. 10–14 days after initial seeding formed colonies were methanol fixed, stained with crystal violet and counted. Surviving fraction at 2 Gy (SF2) was calculated as the fraction of cells surviving 2 Gy compared with non-irradiated cells.

### Neutral comet assay

Cells were treated with indicated times doses of MMAE, mertansine, cetuximab, cetuximab-MMAE, trastuzumab, trastuzumab-MMAE or T-DM1 overnight, and then irradiated with 6 Gy. Cells were collected 15 min post IR, suspended in agarose gel and lysed per assay directions (Trevigen). Samples underwent electrophoresis under neutral conditions and were then stained with Sybr Green. Comet tails were counted in multiple fields (>60 cells per sample) and analysed using CometScore (TriTek Corp). Comet tail length was normalized to vehicle-treated, non-irradiated cells.

### Immunoblotting

Cells were collected and lysed in RIPA buffer (20 mM Tris pH 8, 150 mM NaCl, 5 mM EDTA, 1% Triton X-100) with protease and phosphatase inhibitors (Complete Protease Inhibitor Cocktail and Phos-Stop, Roche). Lysate protein was quantitated by BCA technique (Pierce). Twenty micrograms of lysate underwent electrophoresis using 4–12% Bis-Tris gels (Life Technologies), transferred to PVDF membranes (iBlot) and incubated with indicated primary antibodies HER2, GAPDH (Cell Signaling Technology, catalogue numbers 2242 and 2118) and EGFR (Millapore, catalogue number 06-847) at dilutions of 1:1,000, 1:3,000 and 1:3,000, respectively. Blots were developed by ECL (Pierce). Uncropped blots are shown in relevant Supplementary Figures.

### Immunohistochemistry

Tissue sections were cut from blocks of formalin-fixed paraffin embedded xenografts. Four micron thick tissue sections were stained with antibodies to phospho-histone H3 (pH3, Abcam, catalogue number ab32107) and cleaved caspase-3 (ClC3, Cell Signaling Technology, catalogue number 9661) and used at dilutions of 1:300 and 1:900, respectively. Slides were stained on a Ventana Discovery Ultra (Ventana Medical Systems, Tucson, AZ, USA). Antigen retrieval was performed using CC1 for 24–40 min at 95 °C. The primary antibodies were incubated on the sections for 1 h at 37 °C. Primary antibodies were visualized used DAB as a chromagen using the UltraMap system (Ventana Medical systems) followed by hematoxylin as a counterstain. Slides were rinsed, dehydrated through alcohol and xylene and coverslipped.

### Whole slide scanning and immunostaining quantitation

Immunostained slides were scanned using an Axio Scan.Z1 (Zeiss; Oberkochen, Germany). Axio Scan uses the software Zen2 (Zeiss) for automatic thresholding and tissue detection. The entire tissue section encompassing the xenograft was scanned at 40 × (0.95 numerical aperture) using the default stitching parameters to combine the individual tiles into a single image. Whole-slide images were imported into Definiens Software for quantitative analysis (Definiens; Munich, Germany). Using Definiens Tissue, tumour regions of interest (ROI) were chosen per slide based on the pH3 immunostain. The software uses the color contrast of the DAB and hematoxylin counterstain to determine the basic ROI. Training was done to exclude non-tumour tissue including necrotic regions and mouse stroma. The ROI defined for the pH3 stain was also used to analyse the adjacent section stained for ClC3. For analysis of pH3 (a nuclear stain), the total number of nuclei and the total number of nuclei showing DAB staining (pH3 positive cells) were recorded within each ROI. The percent of positive nuclei was then calculated for each sample. For analysis of ClC3 (a cytoplasmic stain), mean DAB chromagen intensity was measured and calculated within the ROI for each sample.

### *In vivo* tumour xenograft optical imaging

All animal work was done in compliance with the University of California San Diego Institutional Animal Use and Care Committee. Six-to-eight-week-old female athymic nu/nu mice purchased from the University of California San Diego Animal Care Program breeding colony were injected subcutaneously into the bilateral upper thighs with 5 × 10^6^ CAL-27, SQ9-G, SCC-35, SCC-61, A549, HCT-116, OE19 or LN229 tumour cells in a 1:1 Matrigel (BD) and PBS solution. After tumours grew to >100 mm^3^ they were injected with 0.5 nmol of Cy-labelled cetuximab-MMAE, trastuzumab-MMAE (∼4 and ∼3.9 mg kg^−1^, respectively) as described above. For imaging, mice were anaesthetized (1:1 mixture of 100 mg ml^−1^ of ketamine and 5 mg ml^−1^ of midazolam). Animals were imaged using a Maestro Small Animal Imager (CRI) with excitation filter of 620/22 and 645 nm long-pass emission filter with dichroic filter tuned to 670 nm. Imaging was done both with skin on and after skin removal to decrease autofluorescence and scattering. For blood clearance studies, athymic nu/nu mice were anaesthetized with isoflurane and dosed with 5 nmol of the antibody conjugates (∼36.4 trastuzumab and ∼38.6 mg kg^−1^ T-MMAE). At various time points after injection, the tail was pricked and a small volume (5–10 μl) of blood was collected in a heparinized hematocrit tube. Fluorescent images were taken using the Maestro, with the filters mentioned above, and the integrated fluorescent intensity was measured using Image J.

### *In vivo* tumour xenograft experiments

CAL-27, OE19, NCI N87 and HCT116 tumour xenografts were established and tumour growth was measured with digital calipers. Tumour volume was measured blindly to treatment group and calculated using the formula as ½ × length × width^2^. Mice were randomized into groups once the average tumour volume reached >100 mm^3^. Mice were assigned to indicated groups in ‘Results'. MMAE, cetuximab, trastuzumab, C-MMAE, T-MMAE or T-DM1 was intravenously injected in 50 μl. For irradiated mice, the tumour-bearing hindlimbs were focally irradiated while the remainder of the mouse was shielded from IR with custom designed lead blocking >95% of the dose as verified by dosimeters placed on the mouse. Free MMAE was injected on an equimolar basis to C-MMAE in final volume of 3% DMSO. Drug doses and IR fractionation are as indicated in ‘Results'. To prevent unnecessary morbidity, mice were killed if tumour length exceeded 15 mm per protocol. Nonlinear regression least squares fit was used to calculate tumour volume doubling times. Tumour doubling times were extrapolated for T-DM1+IR groups where tumour doubling was been achieved. To minimize the number of mice used, tumours were grown in the bilateral flanks. For CAL-27 tumour xenografts in experiment Fig. 4f, the number of mice and tumours per group were: control (5 mice, 10 tumours), IR (5 mice, 10 tumours), MMAE (4 mice, 8 tumours) MMAE+IR (5 mice, 10 tumours), cetuximab (5 mice, 10 tumours), cetuximab+IR (5 mice, 10 tumours), C-MMAE (4 mice, 8 tumours), C-MMAE+IR (5 mice, 10 tumours). For CAL-27 tumour xenografts in experiment Fig. 4g, the number of mice and tumours per group were: control (4 mice, 8 tumours), IR (5 mice, 10 tumours), cetuximab+MMAE (5 mice, 10 tumours) cetuximab+MMAE+IR (5 mice, 10 tumours), C-MMAE (5 mice, 10 tumours), C-MMAE+IR (5 mice, 10 tumours). For OE19 tumour xenografts in experiment Fig. 5b, the number of mice and tumours per group were: control (5 mice, 10 tumours), trastuzumab (5 mice, 10 tumours) T-MMAE (5 mice, 10 tumours), T-DM1 (5 mice, 10 tumours). For OE19 tumour xenografts in experiment Fig. 5d,e and Table 1, the number of mice and tumours per group were: control (5 mice, 10 tumours), IR (5 mice, 10 tumours), trastuzumab (5 mice, 10 tumours), trastuzumab+IR (5 mice, 10 tumours), T-DM1 (5 mice, 10 tumours) and T-DM1+IR (5 mice, 10 tumours). For NCI N87 tumour xenografts in experiment Fig. 5d,e and Table 1, the number of mice and tumours per group were: control (2 mice, 4 tumours), IR (3 mice, 6 tumours), trastuzumab (3 mice, 6 tumours), trastuzumab+IR (4 mice, 8 tumours), T-DM1 3 mice, 6 tumours) and T-DM1+ IR (4 mice, 8 tumours). For HCT116 tumour xenografts in experiment Fig. 5d,e and Table 1, the number of mice and tumours per group were: control (3 mice, 6 tumours), IR (4 mice, 8 tumours), trastuzumab (3 mice, 6 tumours), trastuzumab+IR (3 mice, 6 tumours), T-DM1 (3 mice, 6 tumours) and T-DM1+ IR (4 mice, 8 tumours).

### Tumour xenograft drug measurement

Tumours were excised, weighed and homogenized in 10 volumes of PBS with a point sonicator (Fisher Scientific) using an amplitude range of 5–15% for a maximum of 20 s while on ice. The homogenates were centrifuged (14*g*, 10 min), then the supernatants were collected and diluted two-fold by addition of 2% acetic acid in acetonitrile, then centrifuged again (14*g*, 10 min). MMAE concentration was determined by LC-MS/MS with Luna-2 C18 column and Agilent Trap XCT mass spectrometer and extracted fragment ion currents at 686.4 and 506.4 were integrated and combined to improve sensitivity.

### Statistical analysis

Unpaired two-sided *t*-tests were performed for IC_50_ and radiosensitization experiments in cell culture. In tumour regression studies, two-way ANOVA analysis was performed with Tukey's multiple comparison group. Survival curves were analysed log-rank. Tumour doubling volume times were analysed by determining 95% confidence intervals. All statistical analyses were performed using Prism software (GraphPad). Statistical analysis for all tumour xenograft data are presented in Supplementary Tables 2–9.

### Data availability

All relevant data are available from the authors.

## Additional information

**How to cite this article:** Adams, S. R. *et al*. Anti-tubulin drugs conjugated to anti-ErbB antibodies selectively radiosensitize. *Nat. Commun.* **7**, 13019 doi: 10.1038/ncomms13019 (2016).

## Supplementary Material

Supplementary Information Supplementary Figures 1-11 and Supplementary Tables 1-9.

## Figures and Tables

**Table 1:** T-DM1 combined with IR increases HER2+ tumor control.

	OE19	NCI N87	HCT116
Control	7 (5–14)	9 (7–16)	3 (2–5)
Trastuz	6 (4–8)	14 (10–21)	4 (2–8)
T-DM1	11 (8–17)	23 (21–25)	3 (2–5)
IR	9 (7–11)	14 (11–20)	6 (4–9)
Trastuz+IR	14 (10–24)	19 (16–22)	7 (5–12)
T-DM1+IR	66 (33+)	113 (82–179)	7 (5–12)

Tumour doubling time in days (mean, 95% CI) of mice in Fig. 4d bearing OE19 or NCI N87 (HER2+) or HCT116 (HER2−) tumour xenografts.
