# Supplementary material for: Anti-tubulin drugs conjugated to anti-ErbB antibodies selectively radiosensitize
Source: Nat Commun. 2016 Oct 4;7:13019. doi: 10.1038/ncomms13019 (PMC5059467; doi:10.1038/ncomms13019)
Supplement: Supplementary Information — Supplementary Figures 1-11 and Supplementary Tables 1-9. [file ncomms13019-s1.pdf]

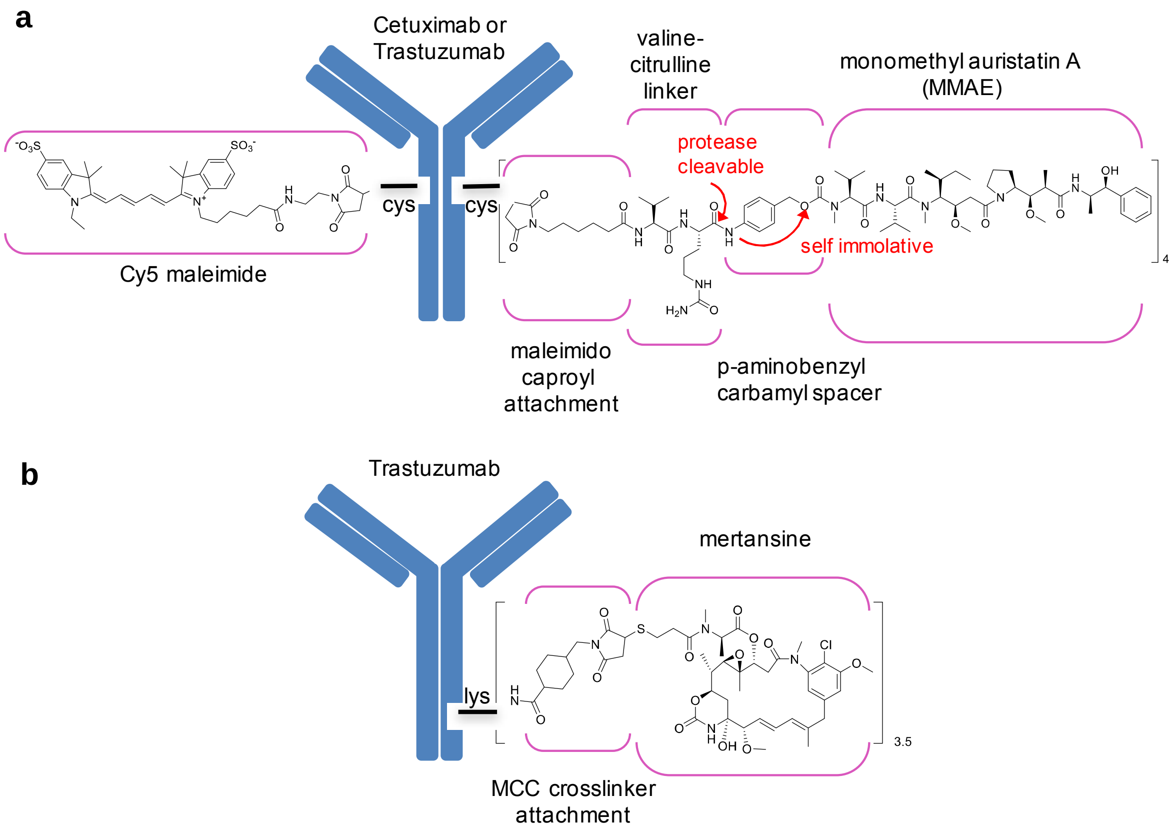

Supplementary Figure 1: Schematics and Chemical structures of radiosensitizer drugs, linkers and attachment groups present in the ADC's used in this study. a) Antibodies to EGFR and Her2 (cetuximab and trastuzumab respectively) conjugated to 4 MC-VC-PABC-MMAE and 1 Cy5 via maleimide reaction with reduced hinge disulfides. Cleavage by lysosomal proteases such as cathepsin D at the amide indicates results in release of MMAE after self-immolative loss of p-quinone imine and CO_2. **b)** DM-1 (ado-trastuzumab emtansine) is composed of a maytansinoid warhead attached by non-cleavable MCC (4-maleimidylcyclohexane-carboxamide) crosslinker to endogenous lysines in trastuzumab. Both A and B contain thioether succinimides that can undergo a retro-Michael addition in plasma and animals resulting in loss of drug from the ADC.

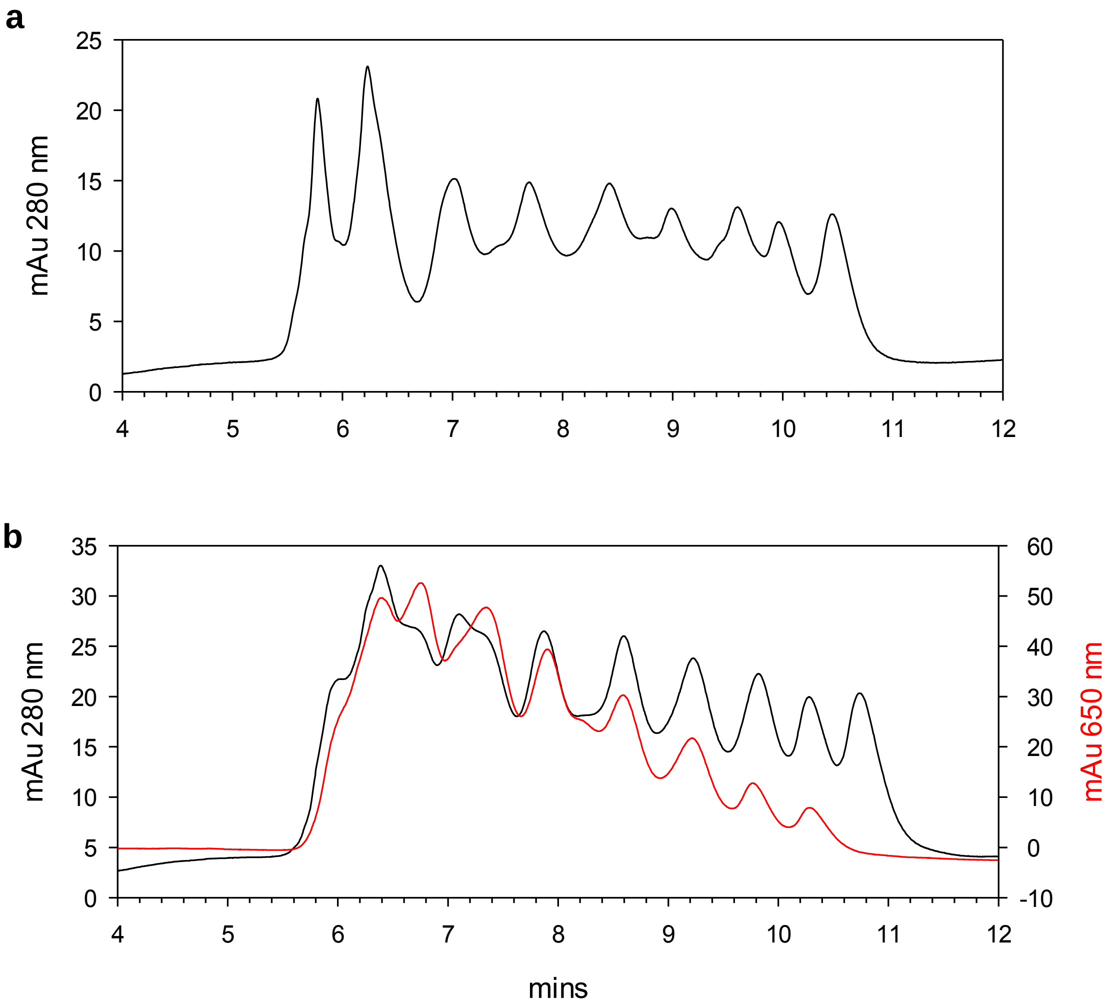

Supplementary Figure 2: Conjugation of MMAE and Cy5 to Cetuximab and Trastuzumab. a) Hydrophobic interaction chromatograms of cetuximab after partial reduction with 4 equivalents of TCEP and subsequent reaction with 4 equivalents of MC-VC-PABC-MMAE and **b)** then 2 equivalents of Cy5-maleimide, followed by gel filtration and concentration.

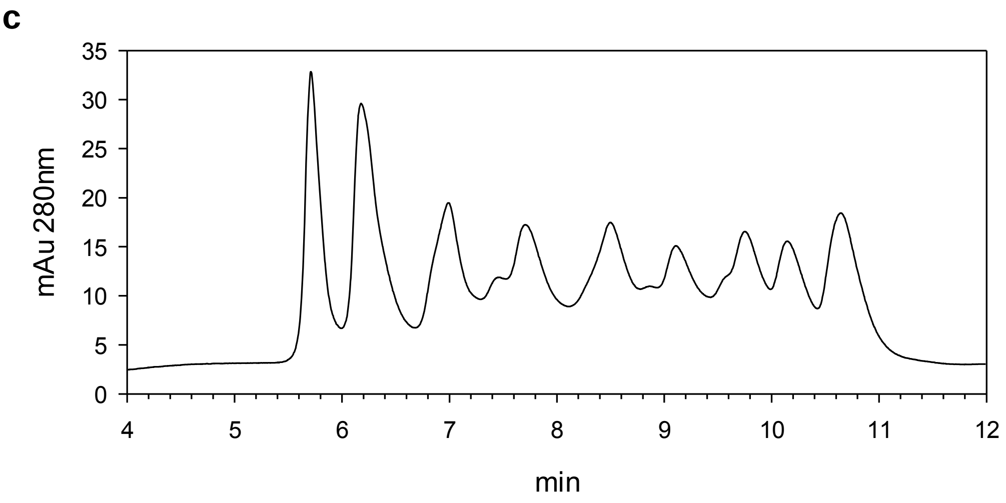

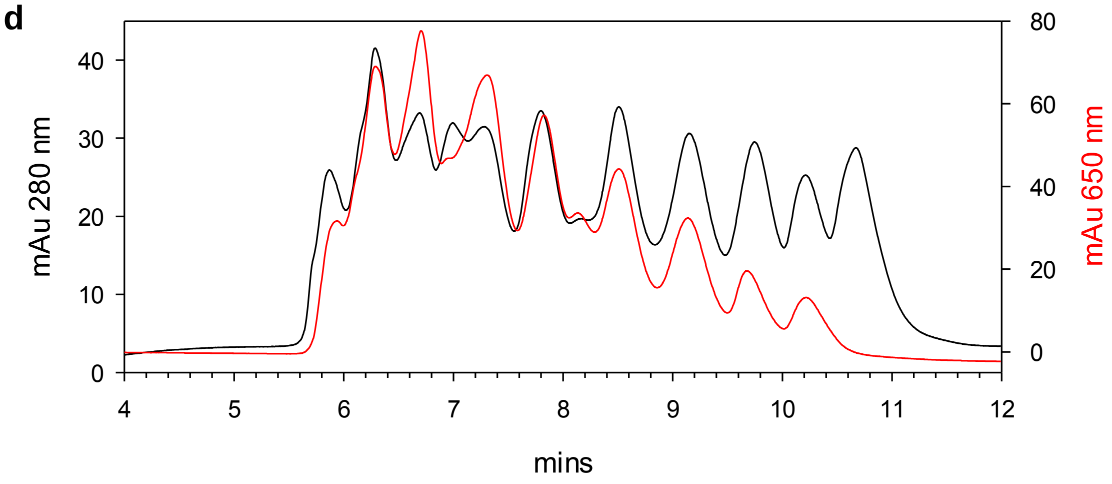

Supplementary Figure 2 (cont): Conjugation of MMAE and Cy5 to Cetuximab and Trastuzumab. c) Hydrophobic interaction chromatograms of trastuzumab after partial reduction with 4 equivalents of TCEP and subsequent reaction with 4 equivalents of MC-VC-PABC-MMAE and then **d)** 2 equivalents of Cy5-maleimide, followed by gel filtration and concentration.

e

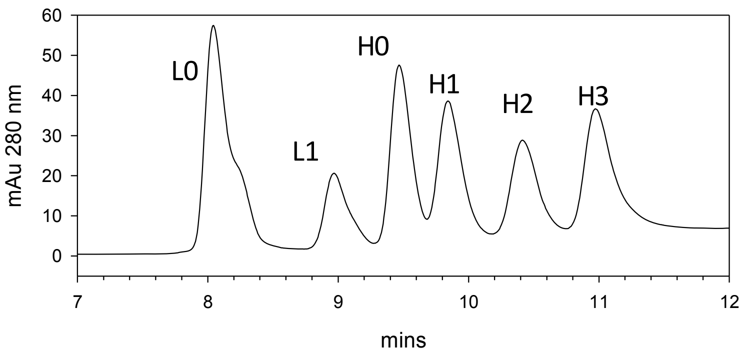

peak	Ave MW (Fnd)	Assignment
L0	23425.3	Light chain (LC)[a]
L1	24742.2 (+1316.9)	LC-MMAE
H0	52763.9 , 52915.2, 51020.6	Heavy chain (HC) isoforms[a]
H1	54080.1 (+1316.2), 52335.0 (+1314.2)	HC-MMAE
H2	55393.8 (+2629.9) , 55556.0 (+2640.8)	HC-$(MMAE)_2$
H3	56712.5 (+3948.6), 56874.3 (+3959.1)	HC-$(MMAE)_3$

f

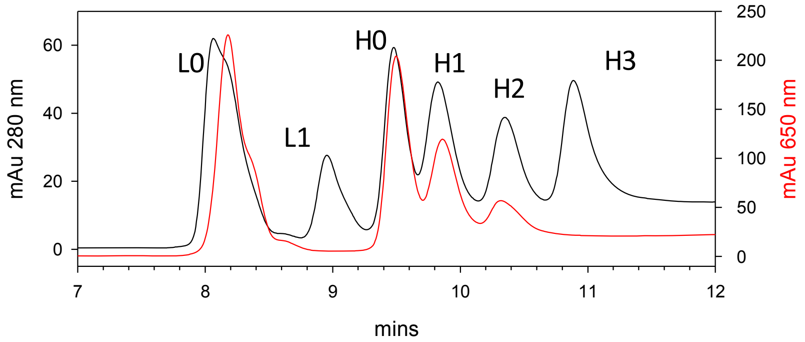

peak	Ave MW (Fnd)	Assignment
L0	23424.2, 24203.6 (+779.4)	LC, LC-Cy5
L1	24741.6 (+1317.4)	LC-MMAE
H0	53541.4 (+777.5), 54472.6 (+1557.4)	HC-Cy5, HC-$(Cy5)_2$
H1	54078.2 (+1316.2), 55017.6 (+2102)	HC-MMAE, HC-MMAE-Cy5
H2	55396.7 (+2632.8) , 55552.0 (+2636.8)	HC-$(MMAE)_2$
H3	56709.6 (+3945.7)	HC-$(MMAE)_3$

Supplementary Figure 2 (cont): Conjugation of MMAE and Cy5 to Cetuximab and Trastuzumab. e) Reverse-phase protein chromatograms and electro-spray mass spectroscopy analysis of Cetuximab after partial reduction with 4 equivalents of TCEP and subsequent reaction with 4 equivalents of MC-VC-PABC-MMAE and **f)** 2 equivalents of Cy5-maleimide, followed by gel filtration and concentration. [a]Cetuximab light chain reported MW is 23426.92 and major heavy chain glycoforms reported MW are 52898.5 and 52975.5 with batch variability. Expected addition in mass is 1316.6 and 779.9 for MC-VC-MMAE and Cy5-maleimide respectively.

g

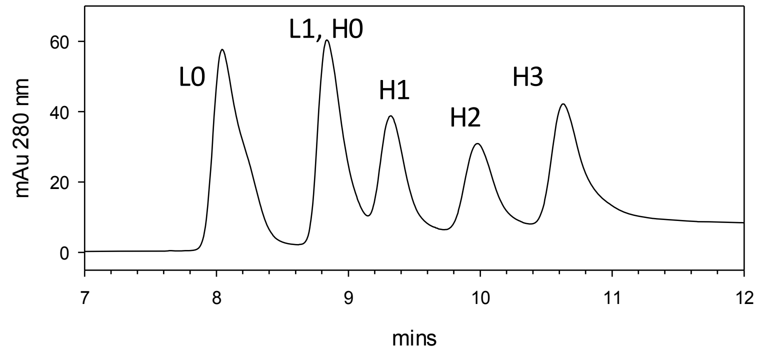

peak	MW (Found)	Assignment
L0	23441.5	Light chain (LC)[a]
L1	24758.4 (+1316.9)	LC-MMAE
H0	50598.0 , 50760.0, 50923	Heavy chain (HC) isoforms[a]
H1	51914.8 (+1314.8), 52076.7 (+1315.9), 52238.4 (+1315.4)	HC-MMAE
H2	53231.7 (+2631.7), 53394.4 (+2634.4)	HC-$(MMAE)_2$
H3	54548.1 (+3950.1), 54710.5 (+3950.9)	HC-$(MMAE)_3$

h

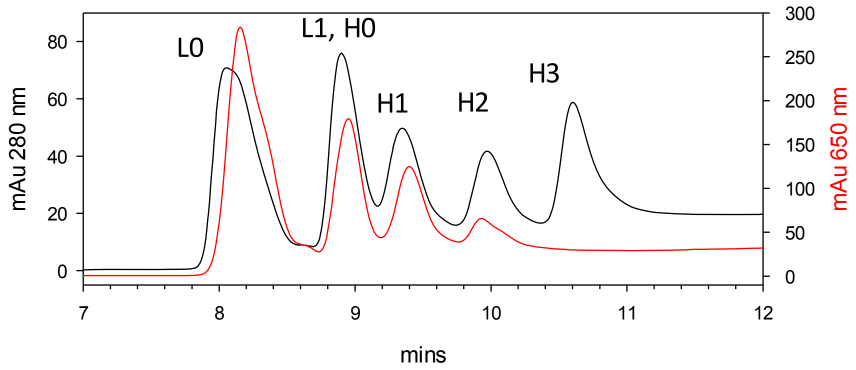

peak	MW (Found)	Assignment
L0	23441.2, 24220.3 (+779.1)	LC, LC-Cy5
L1	24757.7 (+1316.5)	LC-MMAE
H0	50597.5, 51377.8 (+780.3), 51540.0 (+780)	HC, HC-Cy5
H1	51914.9 (+1316.9), 52694.3 (+2096.3), 52076.7(+1316.7)	HC-MMAE, HC-MMAE-Cy5
H2	54011.0 (+ 3413.0) , 53394.1 (+ 2634.1), 53232.1(+2634.1)	HC-$(MMAE)_2$-Cy5, HC-$(MMAE)_2$
H3	54549.3 (+ 3951.3),, 54711.3 (+ 3951.3) 54874.6 (+3951.6)	HC-$(MMAE)_3$

Supplementary Figure 2 (cont): Conjugation of MMAE and Cy5 to Cetuximab and Trastuzumab. g) Reverse-phase protein chromatograms and electro-spray mass spectroscopy analysis of Trastuzumab after partial reduction with 4 equivalents of TCEP and subsequent reaction with 4 equivalents of MC-VC-PABC-MMAE and **h)** 2 equivalents of Cy5-maleimide, followed by gel filtration and concentration. [a]Trastuzumab light chain reported MW is 23443.1 and major heavy chain glycoforms reported MW are 50592, 50754 and 50926 with batch variability (ref). Expected addition in mass is 1316.6 and 779.9 for MC-VC-MMAE and Cy5-maleimide respectively.

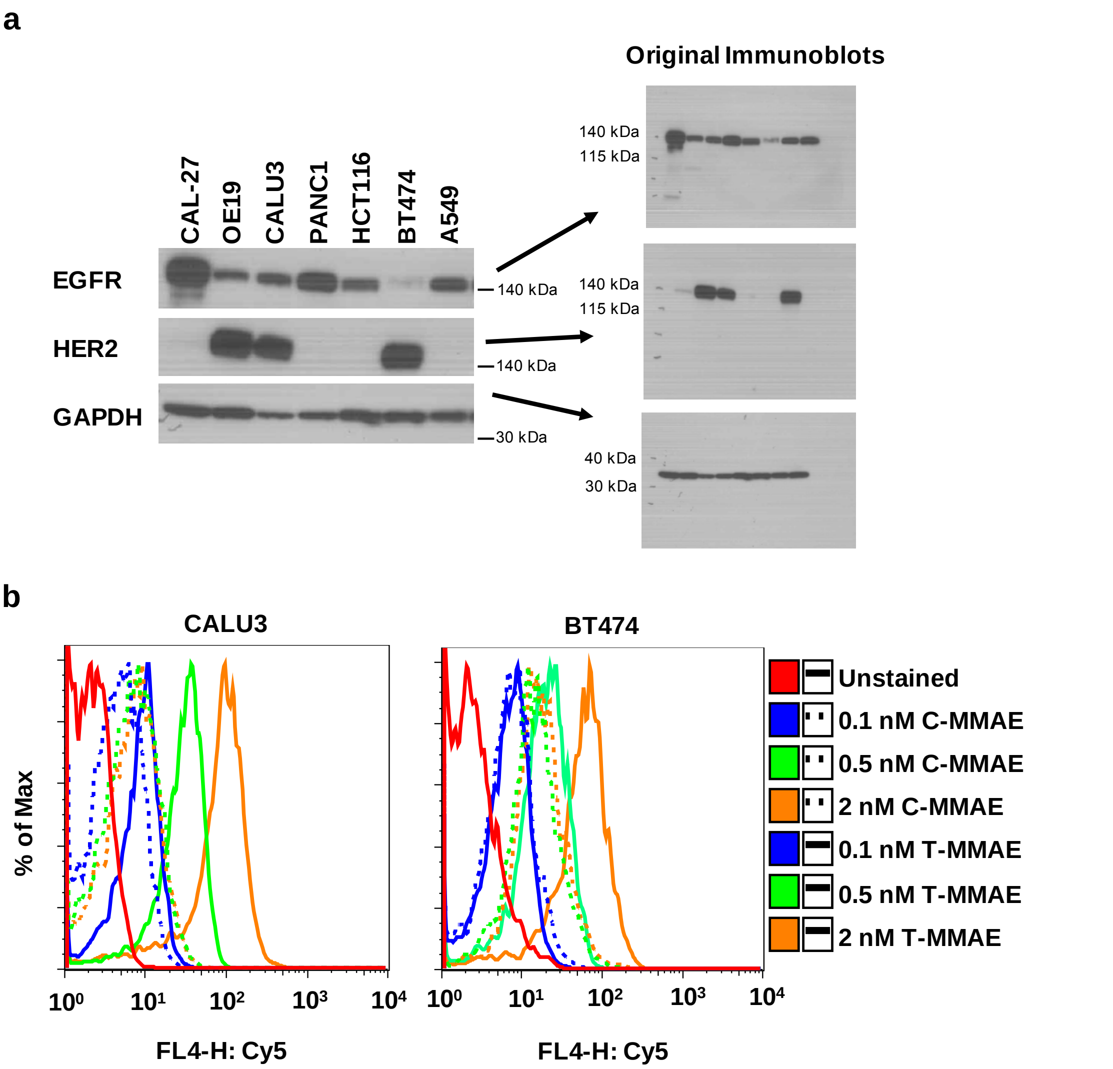

Supplementary Figure 3: EGFR and HER2 expression in cell lines. a) Immunoblot for total EGFR, HER2 and GAPDH in whole cell lysates of indicated cell lines. **b)** Flow cytometry assessment of C-MMAE or T-MMAE cell surface binding. CALU3 and BT474 cells were incubated on ice with increasing concentrations ofCy5 labeled C-MMAE or T-MMAE.

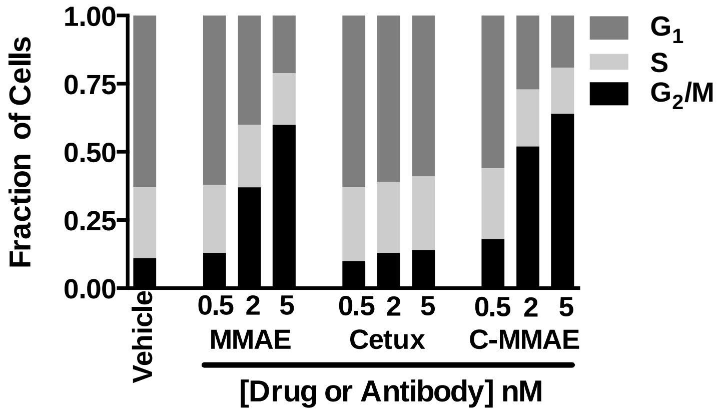

Supplementary Figure 4: MMAE blocks CAL-27 cells in G_2/M as a free drug or when conjugated to cetuximab. Cell cycle profile of CAL-27 cells treated with increasing concentrations of MMAE, cetuximab, or C-MMAE overnight and then stained with propidium iodide.

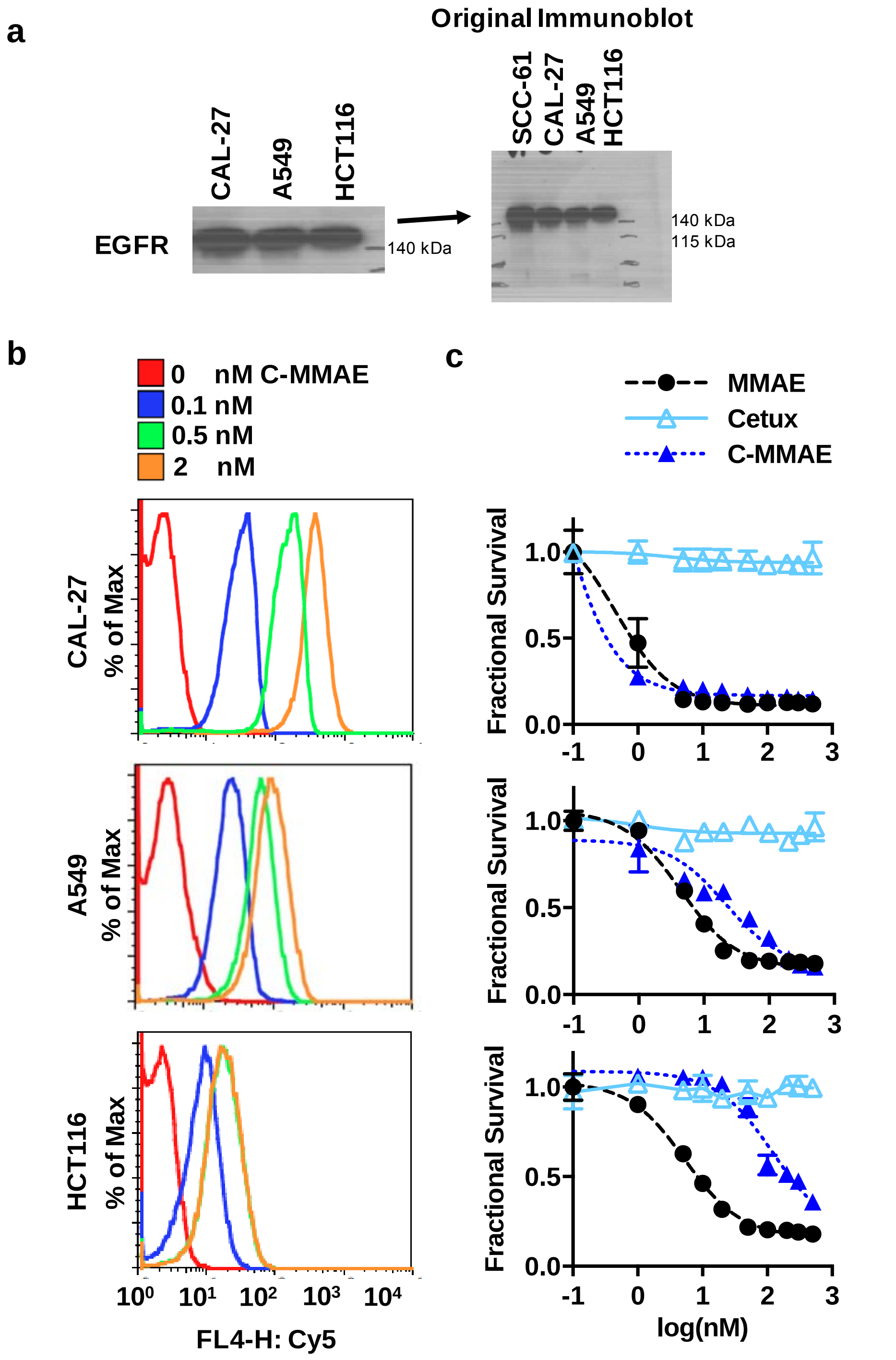

Supplementary Figure 5: Cy5 labeled ADC correlates ErbB receptor availability with ADC toxicity. a) Immunoblot for total EGFR in whole cell lysates from indicated cell lines. **b)** Flow cytometry assessment of C-MMAE cell surface binding. Tumor cells were incubated on ice with increasing concentrations of Cy5 labeled C-MMAE. **c)** Tumor cells were exposed to dose range of MMAE, cetuximab or C-MMAE for 72 hours. Cell viability was measured, normalized to vehicle treated cells and plotted as fractional survival ± SD.

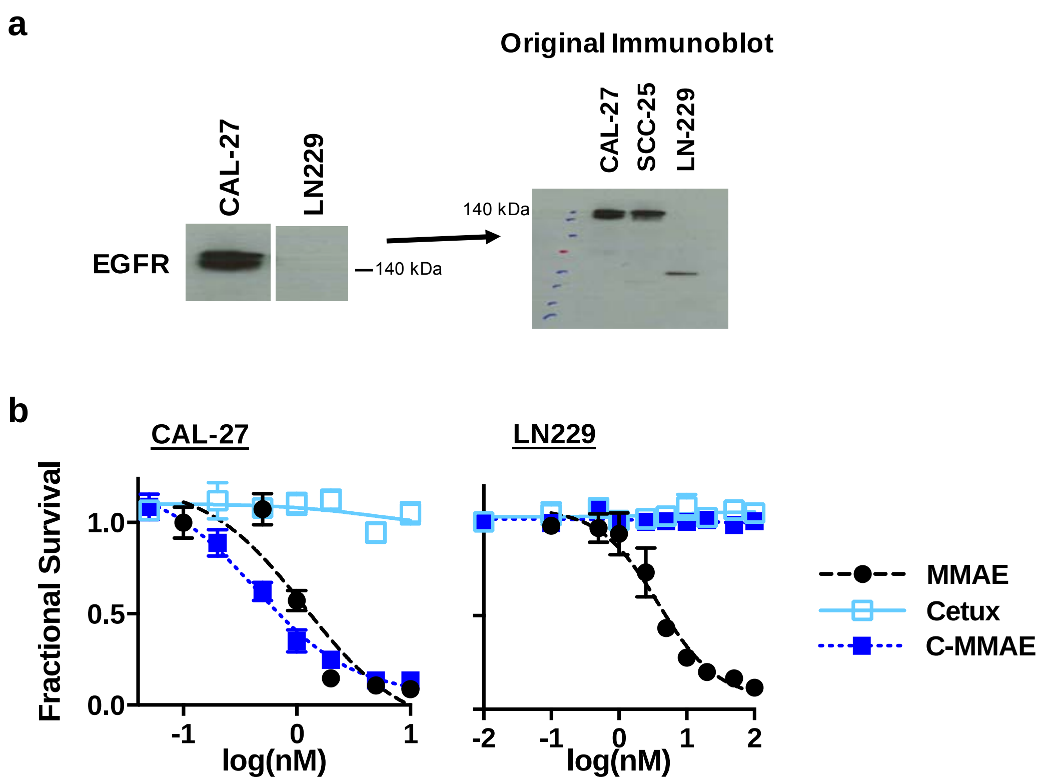

Supplementary Figure 6: a) Immunoblot for total EGFR in whole cell lysates of CAL-27 and LN229 cells. **b)** CAL-27 and LN229 tumor cells were exposed to dose range of MMAE, Cetux or C-MMAE for 72 hours. Cell viability was measured, normalized to vehicle treated cells and plotted as mean fractional survival ± SD.

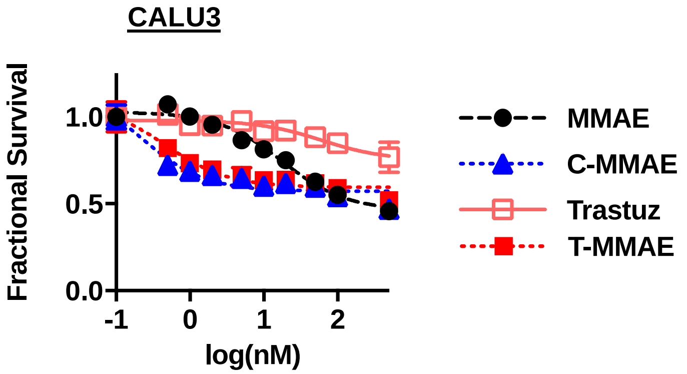

Supplementary Figure 7: CALU3 tumor cells were exposed to dose range of MMAE, ErbB antibody or ErbB ADC for 96 hours. Cell viability was measured, normalized to vehicle treated cells and plotted as mean fractional survival ± SD.

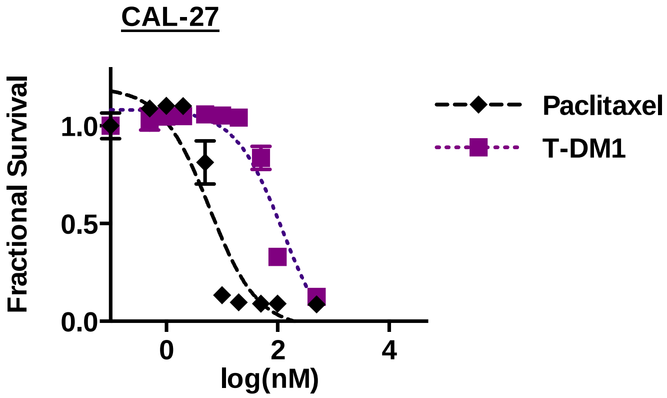

Supplementary Figure 8: CAL-27 cells were exposed to a dose range of paclitaxel or T-DM1 for 96 hours. Cell viability was normalized to vehicle treated cells and plotted as mean fractional survival ± SD.

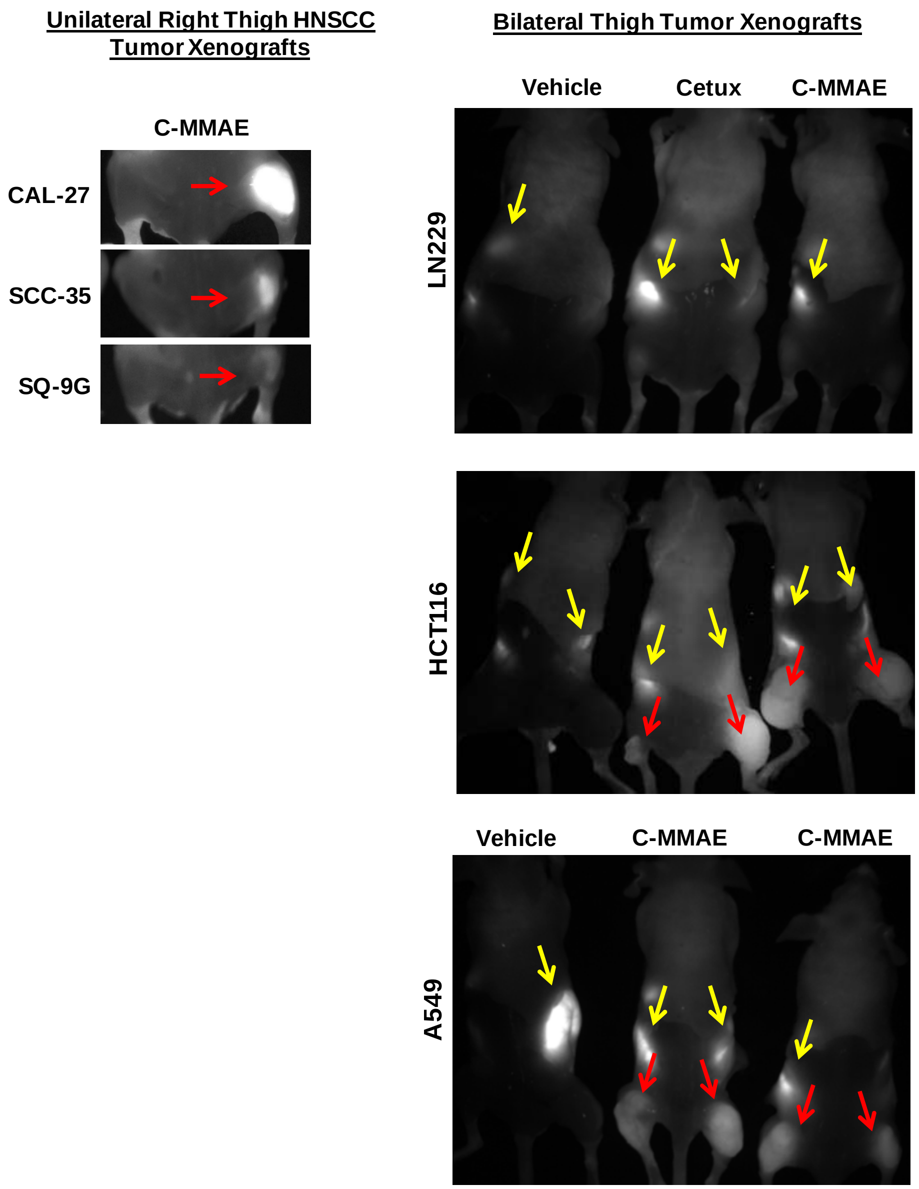

Supplementary Figure 9: C-MMAE accumulates in EGFR expressing tumor xenografts. For HNC tumor xenografts, CAL-27, SCC-35 and SQ-9G tumor cells were only implanted in the right hindlimb. For LN229, HCT-116 and A549 tumor bearing mice, tumors were grown in both the left and right thigh. 0.5 nmoles of Cy5 labeled cetuximab or C-MMAE was IV injected into tumor bearing mice as indicated. Mice were imaged 48 hrs later for Cy5 fluorescence. Red arrows point to tumor Cy5 fluorescence. Yellow arrows point to gut auto-fluorescence.

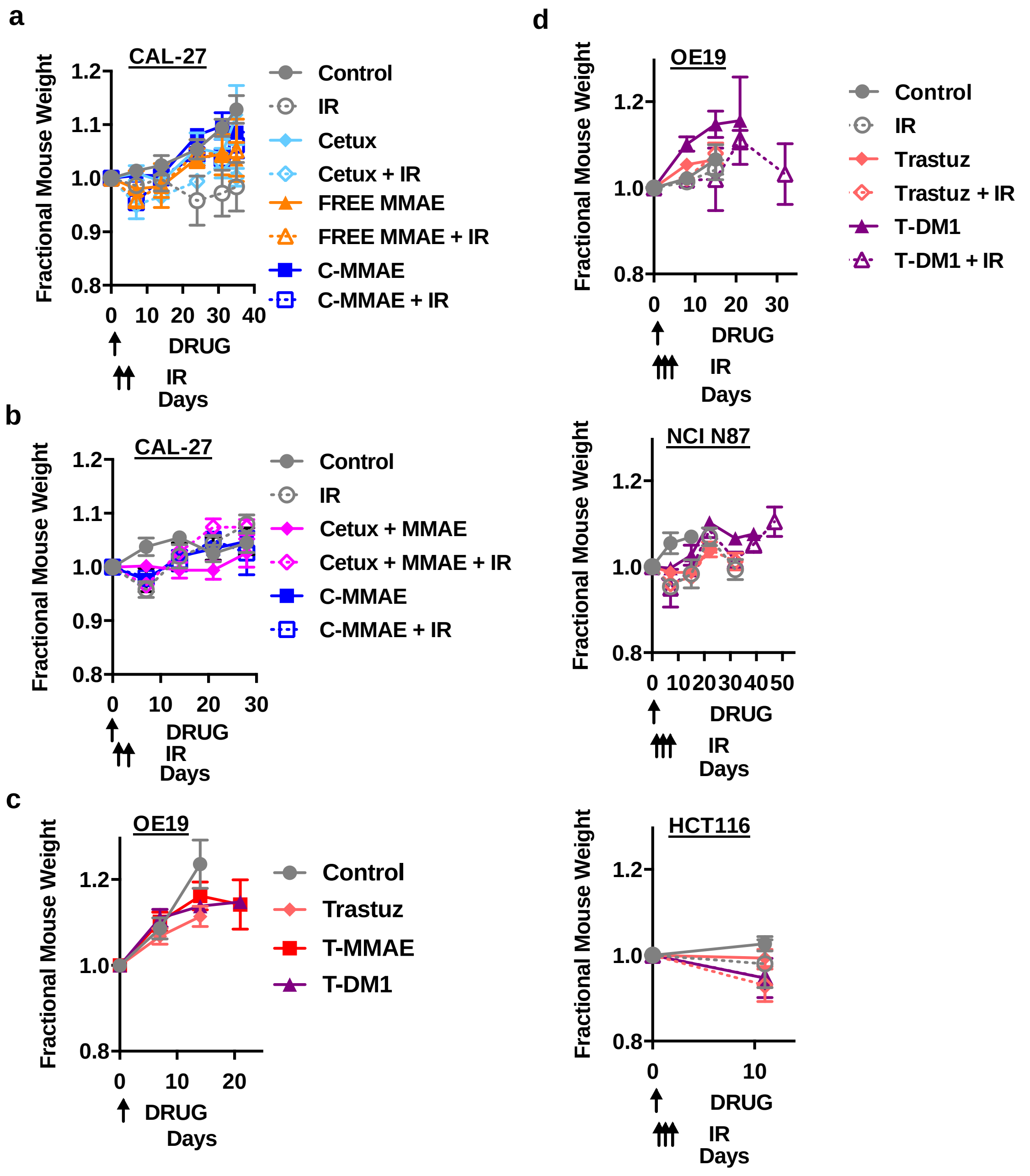

Supplementary Figure 10: Effect on body weight in mice treated with ADC in combination with IR. Individual mouse body weights were normalized to each mouse's weight on initiation of treatment, Day 0. **a)** experiment in Fig 4f. **b)** experiment in Fig 4g. **c)** experiment in Fig 5b. **d)** experiment in Fig 5d. Data is plotted as mean fractional body weight ± SEM.

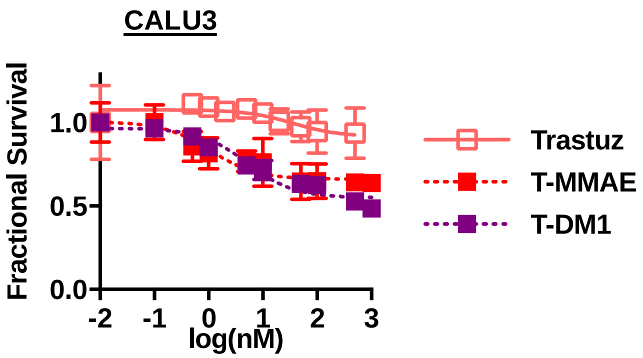

Supplementary Figure 11: CALU3 tumor cells were exposed to dose range of trastuzumab, T-MMAE or T-DM1 for 96 hours. Cell viability was measured, normalized to vehicle treated cells and plotted as mean fractional survival ± SD.

Relative EGFR and HER2 Expression of Cell Lines Used			
	Histology	EGFR	HER2
CAL-27	Head and Neck	+	-
SCC-61	Head and Neck	+	ND
SCC-35	Head and Neck	+	ND
SQ-9G	Head and Neck	+	ND
A549	Non-Small Cell Lung	+	-
CALU3	Non-Small Cell Lung	+	+/-
OE19	Esophageal	-	+
NCI N87	Gastric	ND	+
HCT116	Colorectal	+	-
BT474	Breast	-	+
LN229	Glioma	-	ND

ND: Not Determined

Supplementary Table 1: EGFR and HER2 Status of cell lines used in studies.

Alpha	0.05			
Source of Variation	% of total variation	P value	P value summary	Significant?
Interaction	15.43	<0.0001	****	Yes
Time	46.11	<0.0001	****	Yes
Treatment	26.78	<0.0001	****	Yes

Tukey's multiple comparisons test	Mean Diff.	95.00% CI of diff.	Significant?	Summary
Control vs. IR	325.1	218 to 432.2	Yes	****
Control vs. FREE MMAE	272	158.4 to 385.6	Yes	****
Control vs. FREE MMAE + IR	246.7	139.6 to 353.8	Yes	****
Control vs. Cetux	500.9	393.8 to 608	Yes	****
Control vs. Cetux + IR	559.5	452.4 to 666.6	Yes	****
Control vs. C-MMAE	646.7	533.1 to 760.3	Yes	****
Control vs. C-MMAE + IR	893.5	786.4 to 1001	Yes	****
IR vs. FREE MMAE	-53.13	-166.7 to 60.5	No	ns
IR vs. FREE MMAE + IR	-78.4	-185.5 to 28.72	No	ns
IR vs. Cetux	175.8	68.68 to 282.9	Yes	****
IR vs. Cetux + IR	234.4	127.3 to 341.5	Yes	****
IR vs. C-MMAE	321.6	208 to 435.2	Yes	****
IR vs. C-MMAE + IR	568.4	461.3 to 675.5	Yes	****
FREE MMAE vs. FREE MMAE + IR	-25.28	-138.9 to 88.35	No	ns
FREE MMAE vs. Cetux	228.9	115.3 to 342.5	Yes	****
FREE MMAE vs. Cetux + IR	287.5	173.9 to 401.1	Yes	****
FREE MMAE vs. C-MMAE	374.8	255 to 494.5	Yes	****
FREE MMAE vs. C-MMAE + IR	621.5	507.9 to 735.1	Yes	****
FREE MMAE + IR vs. Cetux	254.2	147.1 to 361.3	Yes	****
FREE MMAE + IR vs. Cetux + IR	312.8	205.7 to 419.9	Yes	****
FREE MMAE + IR vs. C-MMAE	400	286.4 to 513.6	Yes	****
FREE MMAE + IR vs. C-MMAE + IR	646.8	539.7 to 753.9	Yes	****
Cetux vs. Cetux + IR	58.6	-48.52 to 165.7	No	ns
Cetux vs. C-MMAE	145.8	32.2 to 259.4	Yes	**
Cetux vs. C-MMAE + IR	392.6	285.5 to 499.7	Yes	****
Cetux + IR vs. C-MMAE	87.23	-26.4 to 200.8	No	ns
Cetux + IR vs. C-MMAE + IR	334	226.9 to 441.1	Yes	****
C-MMAE vs. C-MMAE + IR	246.8	133.2 to 360.4	Yes	****

Supplementary Table 2: 2 Way ANOVA Analysis with Tukey's Multiple Comparisons Testing. Group comparisons and P value significance testing done with GraphPad Prism. Analysis of experiment shown in Figure 4f. Top table is 2 way ANOVA. Bottom table is Tukey's Multiple Comparisons Testing on day 35. ns for P > 0.05,* P ≤ 0.05, ** P ≤ 0.01, *** P ≤ 0.001, **** P ≤ 0.0001.

Source of Variation	% of total variation	P value	P value summary	Significant?
Interaction	16.26	<0.0001	****	Yes
Time	39.43	<0.0001	****	Yes
Treatment	20.44	<0.0001	****	Yes

Tukey's multiple comparisons test	Mean Diff.	95.00% CI of diff.	Significant?	Summary
Control vs. IR	229.5	142.2 to 316.8	Yes	****
Control vs. Cetux + MMAE	270	182.7 to 357.3	Yes	****
Control vs. Cetux + MMAE + IR	353.5	266.2 to 440.8	Yes	****
Control vs. C-MMAE	347.3	260 to 434.6	Yes	****
Control vs. C-MMAE + IR	456.9	369.6 to 544.2	Yes	****
IR vs. Cetux + MMAE	40.5	-41.8 to 122.8	No	ns
IR vs. Cetux + MMAE + IR	124	41.7 to 206.3	Yes	***
IR vs. C-MMAE	117.8	35.5 to 200.1	Yes	***
IR vs. C-MMAE + IR	227.4	145.1 to 309.7	Yes	****
Cetux + MMAE vs. Cetux + MMAE + IR	83.5	1.199 to 165.8	Yes	*
Cetux + MMAE vs. C-MMAE	77.3	-5.001 to 159.6	No	ns
Cetux + MMAE vs. C-MMAE + IR	186.9	104.6 to 269.2	Yes	****
Cetux + MMAE + IR vs. C-MMAE	-6.2	-88.5 to 76.1	No	ns
Cetux + MMAE + IR vs. C-MMAE + IR	103.4	21.1 to 185.7	Yes	**
C-MMAE vs. C-MMAE + IR	109.6	27.3 to 191.9	Yes	**

Supplementary Table 3: 2 Way ANOVA Analysis with Tukey's Multiple Comparisons Testing. Group comparisons and P value significance testing done with GraphPad Prism. Analysis of experiment shown in Figure 4g. Top table is 2 way ANOVA. Bottom table is Tukey's Multiple Comparisons Testing on day 28. ns for P > 0.05,* P ≤ 0.05, ** P ≤ 0.01, *** P ≤ 0.001, **** P ≤ 0.0001.

Source of Variation	% of total variation	P value	P value summary	Significant?
Interaction	12.08	<0.0001	****	Yes
Time	21.74	<0.0001	****	Yes
Treatment	12.83	0.0030	**	Yes

Tukey's multiple comparisons test	Mean Diff.	95.00% CI of diff.	Significant?	Summary
Control vs. Trastuz	60.7	-228.5 to 349.9	No	ns
Control vs. T-DM1	645.1	355.9 to 934.3	Yes	****
Control vs. T-MMAE	677	387.8 to 966.2	Yes	****
Trastuz vs. T-DM1	584.4	295.2 to 873.6	Yes	****
Trastuz vs. T-MMAE	616.3	327.1 to 905.5	Yes	****
T-DM1 vs. T-MMAE	31.9	-257.3 to 321.1	No	ns

Supplementary Table 4: 2 Way ANOVA Analysis with Tukey's Multiple Comparisons Testing. Group comparisons and P value significance testing done with GraphPad Prism. Analysis of experiment shown in Figure 5b. Top table is 2 way ANOVA. Bottom table is Tukey's Multiple Comparisons Testing on day 14. ns for $P > 0.05$,* $P \leq 0.05$, ** $P \leq 0.01$, *** $P \leq 0.001$, **** $P \leq 0.0001$.

OE19	Control	IR	Trastuzumab	Trastuzumab + IR	T-DM1	T-DM1 + IR
Exponential growth equation						
Best-fit values						
Y0	152.3	126.7	138.8	163.9	155	158.8
k	0.0935	0.08063	0.1211	0.04951	0.06469	0.01045
Tau	10.69	12.4	8.26	20.2	15.46	95.72
Doubling Time	7.413	8.597	5.726	14	10.72	66.35
Std. Error						
Y0	40.9	23.52	31.98	28.98	34.45	26.84
k	0.0215	0.01004	0.01762	0.01043	0.0125	0.00534
95% CI (asymptotic)						
Y0	70.03 to 234.5	79.78 to 173.7	74.5 to 203.1	106 to 221.7	86.26 to 223.7	105.6 to 212.1
k	0.05028 to 0.1367	0.0606 to 0.1007	0.08564 to 0.1565	0.02869 to 0.07033	0.03974 to 0.08963	-0.0001428 to 0.02104
Tau	7.314 to 19.89	9.935 to 16.5	6.39 to 11.68	14.22 to 34.86	11.16 to 25.16	47.54 to +infinity
Doubling Time	5.07 to 13.79	6.886 to 11.44	4.43 to 8.094	9.856 to 24.16	7.733 to 17.44	32.95 to +infinity
Goodness of Fit						
Degrees of Freedom	48	68	48	68	68	104
R square	0.3443	0.5608	0.5858	0.2873	0.3293	0.03303
Absolute Sum of Squares	2604319	2202448	2166354	2047959	3619098	4128537
Sy.x	232.9	180	212.4	173.5	230.7	199.2

NCI N87	Control	IR	Trastuzumab	Trastuzumab + IR	T-DM1	T-DM1 + IR
Exponential growth equation						
Best-fit values						
Y0	145.7	105	125.6	119.8	126.4	116.5
k	0.07417	0.04901	0.05051	0.03685	0.03076	0.006156
Tau	13.48	20.41	19.8	27.13	32.51	162.4
Doubling Time	9.345	14.14	13.72	18.81	22.54	112.6
Std. Error						
Y0	38.44	18.79	18.92	9.65	5.975	6.38
k	0.01445	0.007074	0.008842	0.002891	0.001411	0.001149
95% CI (asymptotic)						
Y0	66.69 to 224.7	67.25 to 142.7	87.39 to 163.9	100.6 to 139.1	114.5 to 138.3	103.9 to 129.1
k	0.04447 to 0.1039	0.03481 to 0.0632	0.03264 to 0.06838	0.0311 to 0.04261	0.02794 to 0.03357	0.003881 to 0.008431
Tau	9.627 to 22.49	15.82 to 28.73	14.62 to 30.63	23.47 to 32.15	29.79 to 35.79	118.6 to 257.6
Doubling Time	6.673 to 15.59	10.97 to 19.91	10.14 to 21.23	16.27 to 22.29	20.65 to 24.81	82.21 to 178.6
Goodness of Fit						
Degrees of Freedom	26	52	40	78	70	126
R square	0.592	0.5051	0.4873	0.7001	0.89	0.1765
Absolute Sum of Squares	808772	749718	317700	368864	108393	328295
Sy.x	176.4	120.1	89.12	68.77	39.35	51.04

HCT116	Control	IR	Trastuzumab	Trastuzumab + IR	T-DM1	T-DM1 + IR
Exponential growth equation						
Best-fit values						
Y0	71.28	79.75	68.87	85.73	70.81	98.91
k	0.2179	0.1242	0.1936	0.09355	0.2299	0.103
Tau	4.589	8.052	5.166	10.69	4.349	9.71
Doubling Time	3.181	5.581	3.581	7.409	3.015	6.731
Std. Error						
Y0	29.45	23.21	34.29	18.52	27.99	29.29
k	0.04035	0.02215	0.04931	0.01729	0.03837	0.0233
95% CI (asymptotic)						
Y0	10.2 to 132.4	32.78 to 126.7	-4.671 to 142.4	47.79 to 123.7	12.77 to 128.9	39.61 to 158.2
k	0.1342 to 0.3016	0.07935 to 0.169	0.08782 to 0.2993	0.05813 to 0.129	0.1503 to 0.3095	0.05582 to 0.1501
Tau	3.316 to 7.45	5.916 to 12.6	3.341 to 11.39	7.754 to 17.2	3.231 to 6.651	6.66 to 17.91
Doubling Time	2.298 to 5.164	4.1 to 8.735	2.316 to 7.893	5.375 to 11.92	2.24 to 4.61	4.617 to 12.42
Goodness of Fit						
Degrees of Freedom	22	38	14	28	22	38
R square	0.7282	0.5146	0.6755	0.5445	0.7777	0.392
Absolute Sum of Squares	607471	750490	275907	187099	621350	935559
Sy.x	166.2	140.5	140.4	81.74	168.1	156.9

Supplementary Table 5: Tumor Doubling Time Statistical Analysis. Nonlinear regression, tumor doubling time and 95% confidence intervals determined with GraphPad Prism. Analysis of data shown in Table 1, OE19, NCI N87 and HCT116 tumor xenografts.

Source of Variation	% of total variation	P value	P value summary	Significant?
Interaction	8.849	<0.0001	****	Yes
Time	26.93	<0.0001	****	Yes
Treatment	11.14	0.0100	**	Yes

Tukey's multiple comparisons test	Mean Diff.	95.00% CI of diff.	Significant?	Summary
IR vs. Trastuz + IR	312.2	103.6 to 520.8	Yes	***
IR vs. T-DM1	117.7	-90.87 to 326.3	No	ns
IR vs. T-DM1 + IR	555.6	347 to 764.2	Yes	****
Trastuz + IR vs. T-DM1	-194.5	-403.1 to 14.07	No	ns
Trastuz + IR vs. T-DM1 + IR	243.4	34.83 to 452	Yes	*
T-DM1 vs. T-DM1 + IR	437.9	229.3 to 646.5	Yes	****

Supplementary Table 6: 2 Way ANOVA Analysis with Tukey's Multiple Comparisons Testing. Group comparisons and P value significance testing done with GraphPad Prism. Analysis of experiment shown in Figure 5d, OE19 tumor xenografts. Top table is 2 way ANOVA. Bottom table is Tukey's Multiple Comparisons Testing on day 22. ns for P > 0.05,* P ≤ 0.05, ** P ≤ 0.01, *** P ≤ 0.001, **** P ≤ 0.0001.

Source of Variation	% of total variation	P value	P value summary	Significant?
Interaction	13.94	<0.0001	****	Yes
Time	36.66	<0.0001	****	Yes
Treatment	20.07	0.0003	***	Yes

Tukey's multiple comparisons test	Mean Diff.	95.00% CI of diff.	Significant?	Summary
IR vs. Trastuz + IR	158.5	63.92 to 253.1	Yes	***
IR vs. T-DM1	203.8	102.7 to 304.9	Yes	****
IR vs. T-DM1 + IR	397.5	302.9 to 492.1	Yes	****
Trastuz + IR vs. T-DM1	45.33	-49.25 to 139.9	No	ns
Trastuz + IR vs. T-DM1 + IR	239	151.4 to 326.6	Yes	****
T-DM1 vs. T-DM1 + IR	193.7	99.09 to 288.2	Yes	****

Supplementary Table 7: 2 Way ANOVA Analysis with Tukey's Multiple Comparisons Testing. Group comparisons and P value significance testing done with GraphPad Prism. Analysis of experiment shown in Figure 5d, NCI N87 tumor xenografts. Top table is 2 way ANOVA. Bottom table is Tukey's Multiple Comparisons Testing on day 32. ns for P > 0.05,* P ≤ 0.05, ** P ≤ 0.01, *** P ≤ 0.001, **** P ≤ 0.0001.

Source of Variation	% of total variation	P value	P value summary	Significant?
Interaction	1.986	0.5514	ns	No
Time	45.02	<0.0001	****	Yes
Treatment	1.934	0.5095	ns	No

Tukey's multiple comparisons test	Mean Diff.	95.00% CI of diff.	Significant?	Summary
IR vs. Trastuz + IR	174.2	1.326 to 347	Yes	*
IR vs. T-DM1 + IR	67.63	-92.39 to 227.6	No	ns
Trastuz + IR vs. T-DM1 + IR	-106.5	-279.4 to 66.3	No	ns

Supplementary Table 8: 2 Way ANOVA Analysis with Tukey's Multiple Comparisons Testing. Group comparisons and P value significance testing done with GraphPad Prism. Analysis of experiment shown in Figure 5d, HCT116 tumor xenografts. Top table is 2 way ANOVA. Bottom table is Tukey's Multiple Comparisons Testing on day 15. ns for P > 0.05,* P ≤ 0.05, ** P ≤ 0.01, *** P ≤ 0.001, **** P ≤ 0.0001.

**Comparison of Survival Curves**	**OE19**	**NCI N87**
Log-rank (Mantel-Cox) test (recommended)		
Chi square	28.51	29.04
df	5	5
P value	<0.0001	<0.0001
P value summary	****	****
Are the survival curves sig different?	Yes	Yes
Logrank test for trend (recommended)		
Chi square	19.94	19.13
df	1	1
P value	<0.0001	<0.0001
P value summary	****	****
Sig. trend?	Yes	Yes
Gehan-Breslow-Wilcoxon test		
Chi square	24.09	25.02
df	5	5
P value	0.0002	0.0001
P value summary	***	***
Are the survival curves sig different?	Yes	Yes

Supplementary Table 9: Survival Curve Statistical Analysis. Group comparisons and P value significance testing done with GraphPad Prism. Analysis of experiment shown in Figure 5e, OE19 and NCI N87 tumor xenografts. ns for P > 0.05,* P ≤ 0.05, ** P ≤ 0.01, *** P ≤ 0.001, **** P ≤ 0.0001.
